# Translocation of (ultra)fine particles and nanoparticles across the placenta; a systematic review on the evidence of in vitro, ex vivo*,* and in vivo studies

**DOI:** 10.1186/s12989-020-00386-8

**Published:** 2020-11-02

**Authors:** Eva Bongaerts, Tim S. Nawrot, Thessa Van Pee, Marcel Ameloot, Hannelore Bové

**Affiliations:** 1 Centre for Environmental Sciences, Hasselt University, Agoralaan Building D, 3590 Diepenbeek, Belgium; 2 Department of Public Health and Primary Care, KU Leuven, Herestraat 49, Box 703, 3000 Leuven, Belgium; 3 Biomedical Research Institute, Hasselt University, Agoralaan Building C, 3590 Diepenbeek, Belgium

**Keywords:** Engineered, (ultra)fine particles, Nanoparticles, Pregnancy, Placenta, Maternal-fetal transfer

## Abstract

Fetal development is a crucial window of susceptibility in which exposure may lead to detrimental health outcomes at birth and later in life. The placenta serves as a gatekeeper between mother and fetus. Knowledge regarding the barrier capacity of the placenta for nanoparticles is limited, mostly due to technical obstacles and ethical issues. We systematically summarize and discuss the current evidence and define knowledge gaps concerning the maternal-fetal transport and fetoplacental accumulation of (ultra)fine particles and nanoparticles. We included 73 studies on placental translocation of particles, of which 21 in vitro/ex vivo studies, 50 animal studies, and 2 human studies on transplacental particle transfer. This systematic review shows that (i) (ultra)fine particles and engineered nanoparticles can bypass the placenta and reach fetal units as observed for all the applied models irrespective of the species origin (i.e.*,* rodent, rabbit, or human) or the complexity (i.e.*,* in vitro, ex vivo, or in vivo), (ii) particle size, particle material, dose, particle dissolution, gestational stage of the model, and surface composition influence maternal-fetal translocation, and (iii) no simple, standardized method for nanoparticle detection and/or quantification in biological matrices is available to date. Existing evidence, research gaps, and perspectives of maternal-fetal particle transfer are highlighted.

**Supplementary Information:**

The online version contains supplementary material available at 10.1186/s12989-020-00386-8.

## Background

Pregnant women and developing embryos/fetuses comprise a particularly vulnerable population, as nanoparticles (NPs) that infiltrate the bloodstream may reach the placenta and possibly the fetus [1]*.* Such in utero exposure may not only influence fetal development and induce adverse pregnancy outcomes, but it can also adversely affect health in later life since the etiology of diseases in adulthood may have a fetal origin [2], as postulated in the Developmental Origins of Health and Disease hypothesis [3]. Various epidemiological studies identified associations between prenatal exposure to (ultra)fine particles and adverse health outcomes (i) at birth including an increased risk of low birth weight (< 2500 g) [4, 5] and preterm birth (< 37 weeks of gestation) [6, 7], and (ii) later in life such as cardiovascular disease [8, 9], respiratory problems [10, 11], and neurodevelopmental alterations [12, 13]. (Ultra)fine particles refer to the particles that are incidentally generated and emitted in the (outdoor) air, often as by-products of fossil fuel combustion or industrial emission. In contrast, NPs are nanosized particles manufactured through controlled engineering processes [14]. Concerning the latter, Manangama et al. showed a significant association between maternal occupational NP exposure and small for gestational age (birth weight < 10th percentile for gestational age) [15]. Appropriately, the question arises if, during pregnancy, particles can translocate from the mother towards the developing fetus. To our knowledge, this is the first systematic review synthesizing all literature regarding the maternal-fetal transfer of (ultra)fine particles and NPs in in vitro*,* ex vivo, and in vivo settings. The systematic review aims to (i) evaluate the translocation of (ultra)fine particles and NPs towards and across the placenta in in vitro cellular barriers, ex vivo placental perfusion models, in vivo animal, and in vivo human studies, (ii) summarize the exploited analytical techniques to determine maternal-fetal NP translocation, and (iii) identify gaps and further research needs.

## Methods

The systematic review was processed according to the Preferred Reporting Items for Systematic reviews and Meta-Analyses (PRISMA) statement [16]. In accordance with the guidelines, our systematic review protocol was published by the International Prospective Register of Systematic Reviews (PROSPERO) on April 28th, 2020 (CRD42020167478, Additional file 1).

### Search strategy

**Fig. 1:**
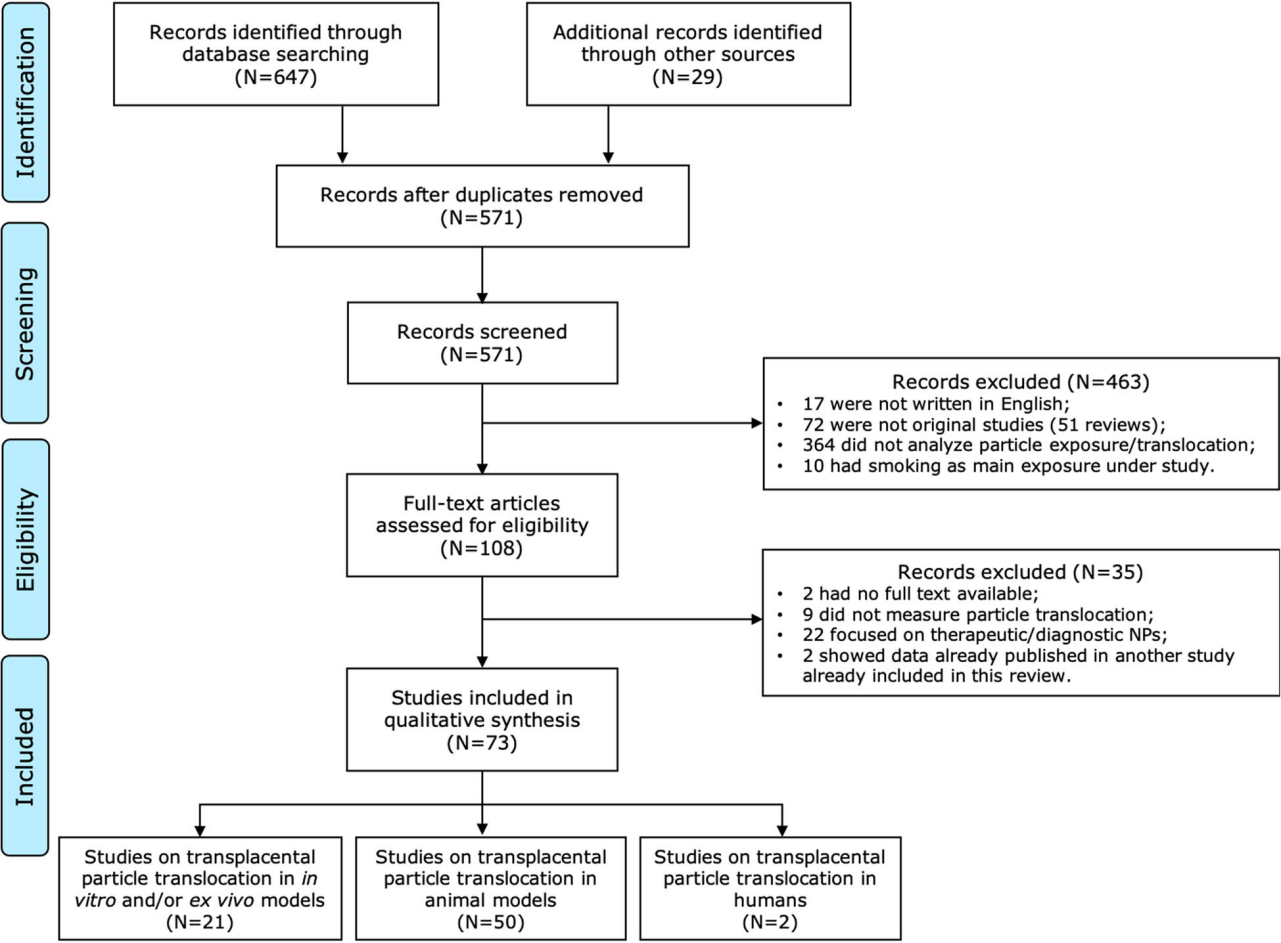
Flowchart, following the PRISMA statement guidelines, of the search strategy used to identify studies examining the maternal-fetal transfer of (ultra)fine particles and NPs. NPs: nanoparticles

The search strategy used to identify relevant studies on the maternal-fetal transfer of (ultra)fine particles and engineered NPs was made up of four stages, as depicted in Fig. 1. In the first stage, articles were identified through a comprehensive literature search using two electronic bibliographic databases: MEDLINE (PubMed interface (www.pubmed.ncbi.nlm.nih.gov)) and Science Citation Index Expanded (Web of Science interface (www.webofknowledge.com/WOS)). The full search strategy was based on the search components “placenta”, “particles”, and “translocation”. Literature search strategies based on Boolean operators were developed using related MeSH terms and text words (Additional file 2). To ensure literature saturation, reference lists in key review papers and included studies were screened to find additional eligible publications that were not retrieved from our initial database searches. The literature search covered articles published in English between January 1st, 1940 and August 11th, 2020.

### Selection criteria

We included human studies and animal studies relevant to human health that addressed the translocation of (ultra)fine particles or NPs across the placenta in an in vitro, ex vivo, and in vivo context. To maintain the focus on air pollutants and engineered nanomaterials in particulate form, we excluded articles examining exposure to tobacco smoke, secondhand smoke, volatile organic components (e.g.*,* benzene, styrene, or xylene) or other volatile substances (e.g.*,* carbon monoxide (CO), ozone (O_3_), nitrogen dioxide (NO_2_), or sulfur dioxide (SO_2_)), and nanomaterials characterized by a high aspect ratio (e.g.*,* nanotubes, nanosheets, or nanowires). Additionally, therapeutic NPs were excluded since these systems are specifically fabricated to (i) achieve targeted and increased placental uptake to treat placental complications (e.g.*,* placenta previa-accreta) [17], (ii) limit transplacental transfer to protect the developing fetus while treating the pregnant mother [18, 19], or (iii) allow transplacental transfer to enable prenatal treatment of congenital diseases (e.g.*,* congenital adrenal hyperplasia or fetal cardiac arrhythmia) while avoiding severe maternal side effects [20, 21]; whereas we want to maintain the focus on unintentional/environmental exposures.

### Selection of studies

Two reviewers (EB and TVP) independently screened the titles and abstracts of all identified papers to exclude studies that did not fulfill one or more of the a priori set inclusion criteria. Any disagreement was resolved through discussion. If no consensus was reached, a third reviewer (HB) was consulted. In the third stage, the full text of selected papers was retrieved and underwent a second screening to see which articles were eligible for inclusion.

### Data extraction process

In the fourth stage, selected studies were grouped according to model and characterized as in vitro, ex vivo, or in vivo (i.e.*,* human or animal) study. The following data were extracted and registered in a predesigned data extraction form: authors, model characteristics, experimental information (e.g.*,* nature of particle exposure, particle size, particle material, surface modification, exposure route, exposure period, and dose), and main findings (e.g.*,* the observation of translocation, and analytical methods used to visualize or quantify particle transfer).

### Synthesis of results

The diversity in, among others, species origin (i.e.*,* rodent, rabbit, or human) or complexity (i.e., in vitro*,* ex vivo, or in vivo) of the applied model, administration route and particle dose, exposure assessment, and analytical detection method, did not allow to carry out a comparative quantitative analysis. Instead, we provided a qualitative overview of the results on the maternal-fetal transfer of (ultra)fine particles and NPs. Narrative result synthesis was achieved via three different steps: (i) summarizing information on the characteristics of included studies in tables per study model, (ii) identifying the maternal-fetal transfer of a given particle, and (iii) grouping those confirmed translocation situations as quantitative or qualitative.

## Results

### Study selection

The initial literature search was completed in January 2020 and re-run in August 2020. A total of 647 articles were identified using PubMed (*N* = 296) and Web of Science (*N* = 351). Additionally, 29 articles were retrieved from the reference list of included reviews (*N* = 18) and other studies (*N* = 11). In total, 105 duplicates were removed using the EndNote software. Titles and abstracts of 571 papers were screened, and 108 full-text papers were assessed for eligibility. Thirty-five articles were excluded because they did not fulfill the following predetermined inclusion criteria; 2 had no full text available, 9 did not measure particle translocation across the placenta, 22 focused on diagnostic/therapeutic NPs, and 2 showed data already published in another included study. The final selection of 73 articles studying transplacental particle transfer included: (i) 21 studies using the ex vivo placental perfusion model and/or in vitro cell line, (ii) 50 animal model studies, and (iii) 2 studies in a human population (Fig. 1).

### Study characteristics

**Fig. 2:**
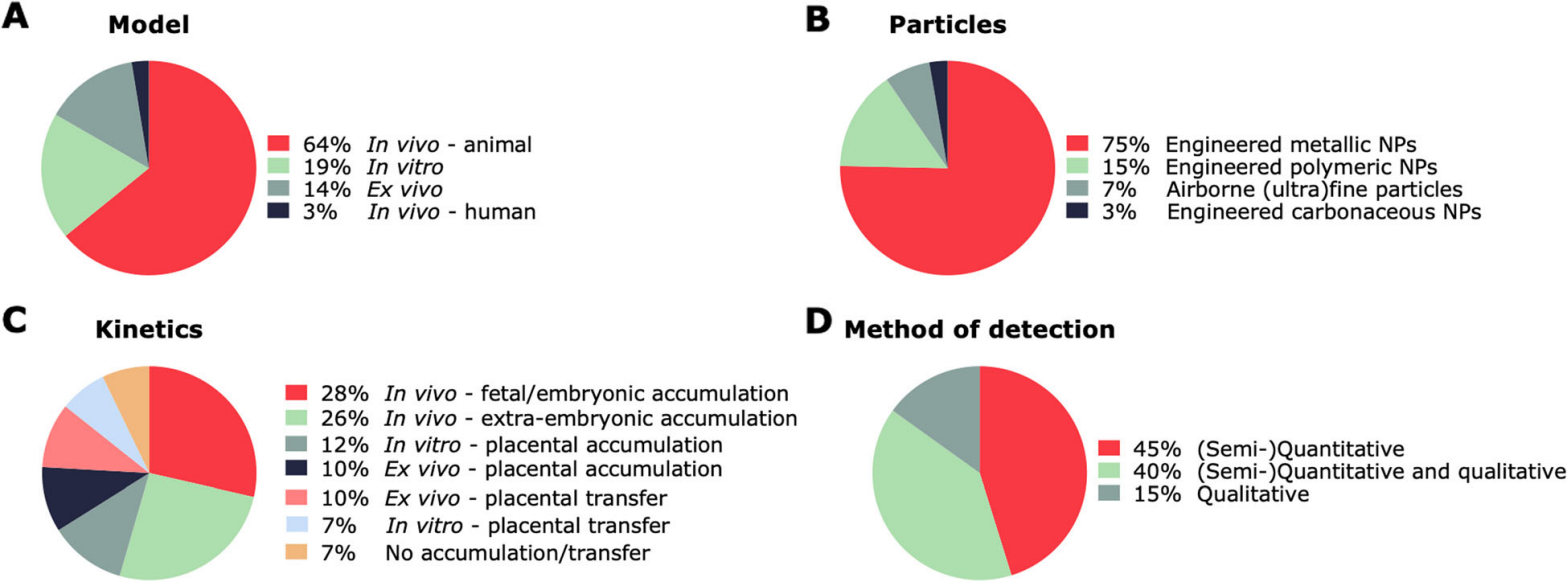
Pie charts describing the characteristics of the included studies. Different models (**a**) were used to assess if (ultra)fine particles and NPs (**b**) can bypass the placenta (**c**) using a variety of detection methods (**d**). NPs: nanoparticles

We have summarized the existing evidence on the maternal-fetal transfer of (ultra)fine particles and NPs in in vitro placental barriers (Table 1), ex vivo placental perfusion models (Table 2), in vivo animal models (Table 3), and humans (Table 4). The characteristics of the included studies are summarized in Fig. 2.

### In vitro maternal-fetal particle transfer

As summarized in Table 1, a total of 15 in vitro cell line studies investigated the maternal-fetal transfer of NPs and (ultra)fine particles. Particle uptake was studied using trophoblast cells grown to confluence on the bottom of a well or Petri dish [29, 30]. Moreover, to study transplacental transfer, cells were cultured on Transwell inserts, which consist of a permeable membrane separating an apical and a basolateral compartment. Transwells were used to study uptake and transplacental transport of NPs across (i) placental trophoblast monolayers of human placental choriocarcinoma (BeWo b30) cells that strongly resemble cytotrophoblast cells (*N* = 6) [22–26, 28], human SV40-transformed trophoblast (3A-sub-E) cells (*N* = 1) [27], or human first trimester trophoblast cells (HTR-8/SVneo (ATCC®, CRL-3271™)) (*N* = 2) [29, 30] (ii) co-cultures of trophoblastic cells (i.e.*,* BeWo b30) and endothelial cells (i.e.*,* primary human placental pericytes (hPC-PL) or human SV40-transformed microvascular placental venous endothelial cells (HPEC-A2) (*N* = 5) [31, 33–36], and (iii) a 3D co-culture of trophoblastic cells (i.e.*,* BeWo b30) and fibroblastic cells (i.e.*,* human villous mesenchymal fibroblasts) (*N* = 1) [32]. Transwell inserts were made from polycarbonate [31, 33–36] or polyethylene terephthalate [34] with a 0.4 μm [22–26, 28] or 3 μm [31, 33–36] pore size. Moreover, a total of 4 studies [31, 34–36] pre-coated the insert with human placental collagen IV to aid cell adherence and growth. Almost all in vitro studies adopted a 24 h NP incubation period, while other studies used 4 h [27], 6 h [24], or 48 h [29, 30] of incubation. The in vitro translocation of 8 different engineered NP types was studied in the included articles: 7 metallic NP types (i.e.*,* silver (Ag), silver sulfide (Ag_2_S), gold (Au), magnetite (Fe_3_O_4_), silicon dioxide (SiO_2_), titanium dioxide (TiO_2_), superparamagnetic iron oxide NPs (SPIONs)), and one polymeric NP type, namely polystyrene (PS) NPs. Moreover, the transplacental in vitro translocation of airborne (ultra)fine particles, namely wood smoke particles [30] and particulate matter ≤2.5 μm (PM_2.5_) [29], was assessed.

Overall, the NP transport from the apical to the basolateral side was limited. Aengenheister et al. showed a translocation success of 0.2 and 1.3% across a BeWo b30 monolayer, 0.1 and 3.6% across an HPEC-A2 monolayer, and 0.1 and 0.6% across the detailed co-culture, respectively for 3 nm poly(ethylene glycol) (PEG)-coated and 4 nm carboxylated Au NPs [31]. Additionally, the same research group reported a negligible transplacental transfer of carboxylated and aminylated TiO_2_ NPs across similar cellular barriers [34]. Multiple studies focused on the effect of particle size and concluded a size-dependent particle transport as larger particles were primarily internalized by the cells with limited transfer to the basolateral compartment [26, 27, 32]. Moreover, surface modification was found to steer particle transport and cell-particle interactions [22, 24, 31–33]. Sodium oleate-coating increased both transcellular transport and cellular uptake, while PEGylation or poly(ethyleneimine) (PEI)-coating resulted in enhanced placental cell association and thus less transport. On the other hand, carboxylation, starch-, or dextran-coating were shown to enhance the transport across the cell barrier while decreasing cell-particle interactions and thus internalization [22, 24, 31–33].

Only 2 studies examined the transfer of ambient air pollution particles across the in vitro placental barrier, more specifically across placental first-trimester trophoblast cells [29, 30]. Both wood smoke particles [30] and PM_2.5_ [29] accumulated in the exposed placental cells as observed by transmission electron microscopy (TEM).

**Table 1:** Basic characteristics of the 15 in vitro studies investigating the maternal-fetal transfer of engineered NPs

Ref	Cell type	Exposure			Detection technique		Main findings
		Particle type/coating or label	Size (nm)	Dose/incubation period	(Semi-) Quantitative	Qualitative	
**Placental trophoblast monolayer**							
[22]	BeWo b30^a^	Ag and Ag_2_S NPs/lipoic acid, citrate, or PEI	28 ± 2, 47 ± 5, 48 ± 5, or 51 ± 5^c^	1 μg/mL/ 4, 6, 18, and 24 h	ICP-MS and spICP-MS	/	Internalization and transfer of different Ag NP types in the BeWo b30 cell layer dependent on surface chemistry.
[23]	BeWo b30	Au NPs/ PEG	10^b^	3.6 × 10^10^ particles/mL/6, 24, and 48 h	/	AMG and TEM	After exposure for 6 h, 24 h, and 48 h, aggregates of Au NPs detected in BeWo b30 cells.
[24]	BeWo b30^a^	Fe_3_O_4_ NPs/Na-oleate	8^b^	50 or 100 μg/mL^e^/ 24 h	/	Bright-field light microscopy and TEM	Increased transport of Fe_3_O_4_ across and uptake in BeWo b30 cells NPs after Na-oleate-coating.
[24]	BeWo b30^a^	SiO_2_ NPs/fluorophore	25 or 50^b^	25 or 50 μg/mL^e^/ 6 h	Fluorescence microscopy	Confocal microscopy	SiO_2_ NPs crossed the BeWo b30 cells without a significant effect of particle size or concentration on transport or internalization.
[25]	BeWo b30^a^	SiO_2_ NPs/fluorophore	25 or 50^b^	100 μg/mL^e^/ 24 h^f^	Fluorescence microscopy	Confocal microscopy	Limited transport of SiO_2_ NPs across the BeWo b30 cells. Confocal microscopy visually confirmed particle accumulation in the cells.
[26]	BeWo b30^a^	PS NPs/fluorophore	50 or 100^b^	500 μg/mL/ 24 h^f^	Fluorescence microscopy	Confocal microscopy	Suggested size-dependent transport and cellular uptake as 50 nm PS NPs transferred to the fetal compartment at a higher rate compared to 100 nm PS NPs.
[27]	3A-sub-E	PS NPs/carboxyl and fluorophore	20, 40, 100, 200, or 500^b^	10 μg/mL/ 4 h	Flow cytometry and confocal microscopy	TEM	Differentially sized fluorescent PS NPs present in the trophoblast cells after 4 h of exposure, Cellular NP uptake highest and lowest for 40 nm and 500 nm PS NPs, respectively.
[28]	BeWo b30^a^	PS NPs/carboxyl and fluorophore	50^b^	10 μg/mL/ 24 h	Fluorescence microscopy	/	Limited translocation of PS NPs across the BeWo b30 cell layer without relation to NP charge.
[29]	HTR-8	PM_2.5_	<2500^d^	0.5 μg/mL/ 24 and 48 h	/	TEM	PM_2.5_ particle uptake visualized within the inner mitochondrial membranes of exposed first trimester placental cells without difference in the 24 h and 48 h exposure groups.
[30]	HTR-8	Wood smoke particles	< 1000^d^	0.5 μg/mL/ 48 h	/	TEM	Wood smoke particles entered the cell and localized to the mitochondria in trophoblast cells, causing structural damage.
**Co-culture**							
[31]	BeWo b30/ HPEC-A2^a^	Au NPs/carboxyl or PEG	3.5 ± 1.2 or 4.5 ± 1.5^c^	25 or 50 μg/mL/ 24 h	ICP-MS	LA-ICP-MS and TEM	PEGylated and carboxylated Au NPs crossed the co-culture in low amounts. Higher cellular uptake for carboxylated Au NPs, and slightly increased translocation for PEGylated Au NPs.
[32]	BeWo b30/ HVMF	Au NPs/carboxyl or PEG	3.5 ± 1.2, 4.5 ± 1.5, 13.5 ± 3, or 14.0 ± 3.5^c^	50 μg/mL/ 24 h	ICP-MS	LA-ICP-MS and TEM	Higher uptake for the smaller, carboxylated Au NPs compared to the larger, PEGylated Au NPs, which barely passed the co-culture.
[33]	BeWo/hPC-PL^a^	SPIONs/starch, PEI, or CMX	n.d.	200 μg/mL/ 3 or 24 h	AAS and MPS	Bright-field light microscopy and confocal microscopy	PEI-coated SPIONs (cationic) remained primarily in the co-culture. Starch- and CMX-coated SPIONS (neutral and anionic, respectively) were able to pass the cell layer to a greater extent.
[34]	BeWo b30/ HPEC-A2^a^	TiO_2_ NPs/ amine or carboxyl	4 to 8^b^	1 μg/mL/ 6 or 24 h	SF-ICP-MS	/	No transplacental transfer of TiO_2_ NPs. Both types internalized in the BeWo b30 and HPEC-A2 cells of the co-culture.
[35]	BeWo b30/ HPEC-A2^a^	PS NPs/ fluorophore	49 or 70^b^	50 or 500 μg/mL/ 24 h^f^	Fluorescence microscopy	/	70 nm PS NPs did not cross the co-culture. Small amounts of 49 nm PS NPs detected in the basolateral compartment after 24 h of exposure. Similar results for static and shaken conditions.
[36]	BeWo b30/ HPEC-A2^a^	PS NPs/ fluorophore and carboxyl	46.3 ± 6.0^c^	10 or 100 μg/mL/ 24 h	AF4-UV	Confocal microscopy	Within 24 h, no transport across the co-culture for both concentrations of PS NPs. Internalization of PS NPs in BeWo cells shown by confocal microscopy.

Data are shown as mean ± standard deviation, ^a^cells grown on Transwell inserts, ^b^primary particle size stated by the manufacturer, ^c^primary particle size determined by TEM, ^d^filter pore size, ^e^0.5 mL apically applied, 1.5 mL basolaterally applied, ^f^incubation under shaken conditions

Abbreviations - *3A-sub-E* human SV40-transformed 3A-Sub-E trophoblast cell line, *AAS* atomic absorption spectrometry, *AF4* asymmetrical flow field-flow fractionation system, *AFM* atomic force microscopy, *Au* gold, *BeWo b30* human placental choriocarcinoma cell line, *CMX* carboxymethyl dextran, *Fe*_*3*_*O*_*4*_ magnetite or iron oxide, *hPC-PL* primary human placental pericytes, *HPEC-A2* human SV40-transformed microvascular placental venous endothelial cells, *HVMF* human villous mesenchymal fibroblasts, *ICP-MS* inductively coupled plasma-mass spectrometry, *LA-ICP-MS* laser ablation-inductively coupled plasma-mass spectrometry, *MPS* magnetic particle spectroscopy, *Na* sodium, *NP* nanoparticle, *PEI* poly(ethyleneimine), *PEG* poly(ethylene glycol), *PM*_*2.5*_ particulate matter smaller than 2.5 μm in diameter, *PS* polystyrene, *SiO*_*2*_ silicon dioxide or silica, *SPIONs* superparamagnetic iron oxide nanoparticles, *TEM* transmission electron microscopy, *TiO*_*2*_ titanium dioxide

### Ex vivo maternal-fetal particle transfer in placental perfusion models

Table 2 gives an overview of the 11 included studies on maternal-fetal particle transfer that employed an ex vivo placental perfusion model to mimic the maternal and fetal blood circulation in the placenta. All placental surrogate models used human term placentae, except for one study that used a rat placenta to investigate transplacental particle transfer [38]. Almost all included studies adopted a 6 h placental perfusion duration (*N* = 10) with the most frequently used NPs being PS (*N* = 4) [40–43] and Au (*N* = 3) [23, 31, 38]. A total of 4 studies combined results from in vitro cell barriers and ex vivo placental perfusion studies to gain additional insights on the kinetics of particles at the placenta [23, 25, 31, 34]. Overall, the ex vivo placental transfer studies reported a substantial decrease in particle concentration in the maternal perfusate over time, without a corresponding increase in the fetal circulation, indicating NP accumulation in the placental tissue. Accordingly, accumulation of Au NPs [23, 31], SiO_2_ NPs [25], and TiO_2_ NPs [34] was observed in the human placenta, both in vitro and ex vivo. Au NPs [31] and SiO_2_ NPs [25] were mainly found in the outer surface of the chorionic villi (i.e.*,* syncytiotrophoblasts), which is in agreement with the observed apical accumulation of the NPs in a BeWo cell monolayer. After 6 h of perfusion, very limited transfer of small (3–4 nm) PEGylated Au NPs [31], and SiO_2_ NPs (25 and 50 nm) [25] was observed in the fetal circulation. For example, after 6 h of perfusion, a placental accumulation of 4–7 μg/g and 2–14 μg/g was observed for 3 nm PEGylated and 4 nm carboxylated Au NPs, respectively, while the respective particle concentration in the fetal perfusate was only 0.0031 μg/mL and 0 μg/mL [31]. As observed for in vitro studies, an association between placental transfer and particle size is present; an increased placental transfer was observed for perfusions with smaller particle sizes [37, 40–43]. For instance, Wick et al. found that PS NPs up to 240 nm were able to cross the placenta and reach the fetal circulation already after a few minutes of perfusion, while 500 nm PS NPs were mainly retained in the placental tissue and maternal circuit [43]. 4 included ex vivo perfusion studies emphasized the effect of surface modification (e.g.*,* PEGylation and carboxylation) on the transplacental passage of engineered NPs [31, 37, 41, 42]. The overall consensus was that PEGylation increases the transplacental particle transport, while carboxylation (e.g.*,* COONa- and COOH-modification) resulted in enhanced placental accumulation. One study also reported increased placental accumulation of aminylated compared to carboxylated TiO_2_ NPs [34].

**Table 2:** Basic characteristics of the 11 ex vivo placental perfusion investigating the maternal-fetal transfer of engineered NPs

Ref	Species	Sample size	Exposure			Detection technique		Main findings
			Particle type/coating or label	Size (nm)	Dose/ perfusion duration	(Semi-) Quantitative	Qualitative	
[37]	Human	6	Ag NPs/ carboxyl or PEG	2–15 or 5–15^b^	40 or 75 μg/mL^c^/ 6 h	spICP-MS	Bright-field light microscopy	Higher transplacental transport for smaller and PEGylated Ag NPs, while carboxylated Ag NPs accumulated more in placental tissue.
[31]	Human	6	Au NPs/ carboxyl or PEG	3.5 ± 1.2 or 4.5 ± 1.5^b^	25 μg/mL^c^/ 6 h	LA- and SF-ICP-MS	/	Only PEGylated Au NPs observed in the fetal circulation. Placental tissue accumulation similar for both Au NP types.
[38]	Rat	11	Au NPs	20^a^	5.8 μg/mL^d^/ 3 h	ICP-MS	Hyper-spectral microscopy imaging	Au NPs translocated across the rat placenta within 20 min of maternal infusion.
[23]	Human	n.d.	Au NPs/ PEG	15 or 30^a^	1.6 × 10^11^ or 1.6 × 10^10^ particles/mL^d^/ 18 min	ICP-MS	TEM and bright-field light microscopy	No placental transfer of Au NPs detected. Visual confirmation of localization of NPs in syncytiotrophoblasts.
[23]	Human	n.d.	Au NPs/ PEG	10 or 15^a^	9.1 × 10^9^ or 2.0 × 10^9^ particles/mL^c^/ 6 h	ICP-MS	TEM and bright-field light microscopy	No transfer of Au NPs across placenta regardless of NP size. Visual confirmation of placental tissue uptake of Au NPs.
[25]	Human	6	SiO_2_ NPs/ fluorophore	25 or 50^a^	100 μg/mL^c^/ 6 h	Fluorescence microscopy	Confocal microscopy	Limited transfer of both SiO_2_ NP sizes to fetal perfusate despite placental accumulation.
[39]	Human	n.d.	Magnetic NPs/ starch or PEI	100 or 150^a^	50 μg/mL^c^/ 6 h	Magnetic system	Bright-field light microscopy	Limited transfer of magnetic NPs from the maternal to the fetal circuit. Histological findings confirmed the presence of NPs in placental tissue.
[34]	Human	6	TiO_2_ NPs/ amine or carboxyl	4 to 8^a^	10 μg/mL^c^/ 6 h	SF-ICP-MS	/	No translocation of both TiO_2_ NP types to the fetal circulation but accumulation in placental tissue.
[40]	Human	7	PS NPs/ fluorophore	80 or 500^a^	25 μg/mL^c^/ 6 h	Fluorescence microscopy	/	80 nm PS NPs able to cross the placenta, while 500 nm PS NPs retained in the placenta or maternal circuit.
[41]	Human	12	PS NPs/ fluorophore and carboxyl	43.7 ± 8, 44.1 ± 7.1, 220.5 ± 5.1, or 289.4 ± 10.2^b^	25 μg/mL^e^/ 6 h	Fluorescence microscopy	TEM	Increased translocation of plain compared to carboxylated PS NPs after 6 h of perfusion. Significantly higher transfer of NPs in the fetal to maternal direction observed with bidirectional transfer studies. Placental accumulation of all NPs regardless of modification and perfusion direction.
[42]	Human	32	PS NPs/ fluorophore, amine, or carboxyl	63 ± 10, 71 ± 11, 78 ± 20, 88 ± 7, 89 ± 3, 181 ± 11, 224 ± 17, 455 ± 32, 451 ± 28, 494 ± 29, or 499 ± 8^b^	25 μg/mL^c^/ 6 h	Fluorescence microscopy	/	Plain and small carboxylated PS NPs but not aminylated PS NPs transferred across the placenta after 6 h of perfusion.
[43]	Human	16	PS NPs/ fluorophore	50, 80, 240, or 500^a^	25 μg/mL^c^/ 6 h	/	TEM	PS NPs up to 240 nm crossed the placenta and reached the fetal circuit. 500 nm PS NPs mainly retained in the placental tissue and maternal circuit.

Data are shown as mean ± standard deviation, ^a^primary particle size stated by the manufacturer, ^b^primary particle size determined by TEM, ^c^re-circulating (closed) dual perfusion system, ^d^open dual perfusion system, ^e^bidirectional perfusion

Abbreviations - *Ag* silver, *Ag*_*2*_*S* silver sulfide, *Au* gold, *ICP-MS* inductively coupled plasma-mass spectrometry, *LA-ICP-MS* laser ablation-inductively coupled plasma-mass spectrometry, *n.d.* not defined, *NP* nanoparticle, *PEI* poly(ethyleneimine), *PEG* poly(ethylene glycol), *PS* polystyrene, *SF-ICP-MS* sector field-inductively coupled plasma-mass spectrometry, *SiO*_*2*_ silicon dioxide or silica, *spICP-MS* inductively coupled plasma-mass spectrometry in single-particle mode, *TEM* transmission electron microscopy, *TiO*_*2*_ titanium dioxide

### In vivo maternal-fetal particle transfer in animal models

**Fig. 3:**
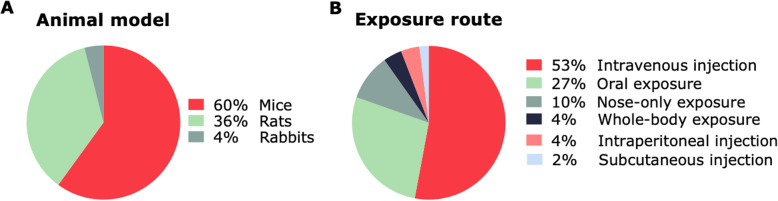
Pie charts describing the characteristics of the animal studies included in the review. In vivo maternal-fetal particle transfer was assessed in animal models (**a**) exposed to (ultra)fine particles and NPs via different methods of administration (**b**). NPs: nanoparticles

In total, 49 animal studies on maternal-fetal particle transfer were included. Forty-three studies showed quantitatively and/or qualitatively that particles reached the placenta after gestational exposure, among which 16 studies observed bioaccumulation of nanosized particles in tissues collected during the embryonic (i.e.*,* organogenesis) and/or fetal period (Table 3). As depicted in Fig. 3a, rodents were the primary animal model used in the included studies (either mouse (*N* = 29) or rat (*N* = 18)), whereas only 2 studies used rabbits as a model for gestational particle translocation [91, 92]. Moreover, the latter are the only studies examining transplacental translocation of airborne (ultra)fine particles, whereas the remainder of in vivo animal studies focused on engineered NPs.

#### Engineered NPs

A total of 48 animal studies examined the transplacental transfer of engineered NPs, including (i) metallic NPs (*N* = 43) (i.e.*,* Ag, alumina (Al_2_O_3_), Au, quantum dots (QDs), cadmium oxide (CdO), cerium dioxide (CeO_2_), Cu (copper), iron oxide (Fe_2_O_3_), platinum (Pt), SiO_2_, TiO_2_, and zinc oxide (ZnO), zirconium dioxide (ZrO_2_) NPs), (ii) carbonaceous NPs (*N* = 2) (i.e.*,* fullerene (C60)), and (iii) polymeric NPs (*N* = 3) (i.e.*,* poly(glycidyl methacrylate) (PGMA), and PS) (Table 3). Fetal/embryonic NP accumulation was observed in 32 of the included animal studies, more specifically NPs were detected in the fetal brain [27, 44, 48, 52, 60, 73, 78, 80, 81, 91], fetal liver [27, 48, 50, 52, 60, 69, 73, 83, 84, 86], fetal lung [27, 48, 52, 75], fetal kidney [48, 52, 84], fetal gastrointestinal tract (GIT) [88], and fetal blood [92]. The preferred method for NP administration was intravenous (i.v.) injection (*N* = 27) or gavage (*N* = 14) (Fig. 3b). The i.v. route is preferred to examine tissue distribution and elimination of NPs while avoiding absorption by the GIT and first-pass elimination in the liver; on the other hand, the oral route is used to assess the ingestion of NPs. Fennell et al. used both i.v. injection and oral administration to expose pregnant rats to 20 nm and 110 nm Ag NPs, and showed increased placental Ag NP levels compared to fetal levels regardless of the administration route [51]. Despite inhalation exposure being the primary route in environmental and occupational settings, only 6 animal studies [47, 66, 68, 77, 91, 92] examined particle translocation following this exposure route. While the studies are limited, the conclusion is unambiguous. All studies except two [68, 77] showed the translocation of Ag NPs [47], CdO NPs [66], and DEPs [91, 92] to the placenta and/or fetus following maternal inhalation exposure. In total, 7 included in vivo animal studies revealed a size- or dose-dependent distribution of NPs from mother to fetus, meaning that smaller particles administered in higher doses tend to accumulate more in placental and embryonic/fetal tissue [27, 46, 55, 57, 62, 73, 85]. For instance, measured cadmium (Cd) levels in mice pups were positively associated with the injected CdTe/CdS QD dose their mothers received. Moreover, a smaller QD size was found to be associated with increased accumulation of Cd in the pups [62]. Also, 1.4, 18, and 80 nm negatively charged Au NPs were found in placentae and amniotic fluid samples of i.v. injected pregnant rats. Only fractions of the initial dose of 1.4 and 18 nm Au NPs reached the fetal tissue, suggesting a size-dependent maternal-fetal translocation of Au NPs [57]. The effect of surface charge on transplacental transfer of engineered NPs was discussed in cited studies [69, 74, 89]. Polymeric NPs coated with PEI (i.e.*,* cationic polymer) preferentially accumulate in the rat placenta over negatively charged polymeric NPs [89] as confirmed by Di Bona et al. who showed an increased placental crossing and consequent elevated fetal levels of PEI-coated Fe_2_O_3_ NPs in i.v. injected pregnant mice as opposed to negatively charged poly(acrylic acid) (PAA)-coated Fe_2_O_3_ NPs [69]. Additional studies on surface composition were included [49, 61, 90]; one showed a reduction in maternal-fetal transfer after chitosan-coating of Ag NPs [49]. Other studies reported a time-dependent accumulation of Ag NPs, CeO_2_ NPs, and fullerenes as they found that the fetal NP concentration increased over time, reached a peak, and then declined [54, 67, 87]. Moreover, critical exposure windows during gestation were evaluated by defining changes in particle transfer after varying the day of NP exposure [60, 61, 70, 71, 87]. Pregnant rats were divided into five groups and differently exposed with radioactively-labeled fullerenes (^14^C(C60)) via single i.v. injection. The experimental groups covered different stages of pregnancy and varied in exposure duration and period until examination. The percentage of radioactivity recovered in rat placenta was higher at later stages of pregnancy (2% recovery at gestational day (GD) 18, compared to 0.05% at GD11). On the other hand, radioactivity detected in fetuses was lower at later stages compared with earlier stages of gestation (0.2% at GD11 and 0.04% at GD18), which can be explained by the lack of a developed placenta during early gestation [87]. Seven animal studies did not find evidence of maternal-fetal transfer of engineered NPs [56, 63, 68, 72, 77, 79, 82].

#### Airborne (ultra)fine particles

Only 2 animal studies examined the maternal-fetal translocation of ambient air pollution particles [91, 92]. Black particles were observed by TEM in the maternal lungs and blood vessels of pregnant rabbits after exposure for 2 h/day, 5 days/week to 1 mg/m^3^ of 69 nm diesel exhaust particles (DEP) from GD3 to 27. Non-aggregated and NP-like particles were detected in the placenta, maternal blood space, trophoblastic cells, and in fetal blood [92] as well as in the fetal olfactory tissue [91]. However, the identification of these particles in the tissues was solely based on TEM observations of black particles without confirming their origin from the diesel aerosols [91, 92].

**Table 3:** Basic characteristics of the 50 animal studies investigating maternal-fetal transfer of ambient (ultra)fine particles and engineered NPs

Ref	Strain/ Species	Sample size	Exposure			Detection technique		Main findings
			Particle type/Coating or label	Size (nm)	Administration route/ dose/ exposure period	(Semi-) Quantitative	Qualitative	
**Metallic NPs**								
[44]	CD-1 mice	18	Al_2_O_3_ NPs	20.9 ± 9.5 or 112.4 ± 24.5^b^	nasal drip/ 0 or 50 mg/kg/ 14 days before mating-PND0^g^	AAS	/	Higher Al levels in hippocampi of pups from mice exposed to Al_2_O_3_ NPs before and during pregnancy compared to control pups.
[45]	CD-1 mice	29	Ag NPs/ citrate	50^b^	i.v./ 0, 1.2, or 2.2 mg/kg/ GD7–9^d^	ICP-MS	TEM and EDX	Distribution of Ag NPs to most maternal organs and extra-embryonic tissues without significant fetal accumulation.
[46]	CD-1 mice	40	Ag NPs/ citrate	10^a^	i.v./ 0 or 2.2 mg/kg/ GD7–9^d^	ICP-MS	Hyperspectral microscopy imaging	No transfer of Ag NPs across the placenta in large amounts but accumulation in the visceral yolk sac and maternal tissue.
[47]	C57Bl/6 mice	12–15	Ag NPs	19.3 ± 2.3^b^	nose-only inhalation/ 0 or 0.64 mg/m^3^ for 1 or 4 h/day/ GD0.5–14.5^e^	spICP-MS and ICP-MS	TEM/EDX	Ag NPs identified and quantified in placenta, yet very low fetal levels.
[48]	Sprague Dawley rats	40	Ag NPs/ citrate	55^b^	oral/ 0, 0.2, 2, or 20 mg/kg/ GD7–20^d^	AAS	/	Higher Ag tissue contents in all treated groups compared to control dams and pups, indicating transplacental Ag NP transfer.
[49]	Wistar rats	12	Ag NPs/ chitosan	19.5 ± 6.72^b^	i.p./ 0 or 100 mg/kg/ GD 6, 8, and 10^d^	AAS	TEM	Coated and plain Ag NPs detected in significantly higher levels in maternal tissues, placenta, and fetuses compared to control rats. Chitosan coating decreased the silver content significantly.
[50]	Wistar Rats	60	Ag NPs/ citrate	20 ± 4^a^	oral/ 0 or 25 mg/kg/ GD1–19^d^	ICP-MS	/	Silver content in the rat offspring’s liver of exposed group differs significantly from control group, suggesting a transplacental transfer of Ag NPs.
[51]	Sprague Dawley rats	36	Ag NPs/ PVP	20 or 110^a^	i.v./ 0 or 1 mg/kg/ GD18^d^ and oral/ 0 or 10 mg/kg/ GD18^d^	ICP-MS	/	Ag NPs measured in the rat placenta and fetuses for both NP sizes. Concentration of Ag NPs in the placenta higher than measured in blood or fetuses for both administration routes.
[52]	Sprague Dawley rats	8	Ag NPs/ citrate	7.9 ± 0.95^b^	oral/ 0 or 250 mg/kg/ 14 days before mating-PND4^d^	ICP-MS	TEM	Accumulation of Ag NPs observed in pups of exposed dams with decreasing concentrations from kidney, lung, liver to brain.
[53]	Wistar rats	7	Ag NPs/ PVP and [^110m^Ag]	34.9 ± 14.8^b^	oral/ 1.69 or 2.21 mg/kg/ GD20^g^	Gamma spectroscopy	/	Ag NPs identified in fetuses of pregnant rats in amounts significantly exceeding the detection limit.
[54]	Wistar rats	30	Ag NPs	4.32 to 16.9^b^	i.v./ 0 or 2 mg/kg/ GD19^d^	ICP-OES	TEM	Time-dependent increase in fetal Ag NP levels, reaching a peak 6 h after injection and showing a decline afterward.
[55]	CD-1 mice	16	Au NPs	19.6 or 49.3^a^	i.v./ 0 or 100 mg/kg/ GD16–17^g^	ICP-MS	AMG	Higher amount of Au NPs in maternal livers and placentae from mice injected with 20 nm compared to 50 nm NPs without detectable levels in fetal organs for both sizes.
[56]	C57Bl/6 mice	13	Au NPs	2 or 40^a^	i.v./ 0, 12.13, or 58.21 mg/mouse/ GD17^g^	/	AMG	No accumulation of both Au NP sizes in fetuses nor placentae.
[57]	Wistar-Kyoto rats	12	Au NPs/S-TPP and [^198^Ag]	1.4, 18, or 80^a^	i.v./ 0.005 or 0.025 mg/rat/ GD18^g^	Gamma spectroscopy	/	All three Au NP sizes found in placenta of pregnant rats. Fractions of 1.4 and 18 nm Au NPs but not 80 nm Au NPs found in the fetuses.
[58]	C57Bl/6 mice	18	Au NPs/ PEG	3, 13, or 30^a^	i.v./ 0.9 mg/kg/ GD17^g^	ICP-MS	TEM	All three Au NP sizes reached the placenta of pregnant mice, but fetal Au NP concentrations were negligible.
[59]	Albino rats	15	Au NPs/ PEG	5.1 ± 0.6 or 32.0 ± 3.6^b^	i.v./ 0 or 0.8 mg/kg/ GD10^g^	AAS	AMG	Both Au NP sizes penetrate the rat placenta. Higher Au NP levels in maternal tissues (e.g., spleen) compared to fetal tissues.
[60]	CD-1 mice	25	Au NPs/ PEG	30^b^	i.v./ 0 or 5 mg/kg/ GD5.5–7.5 and 11.5–13.5^e^	ICP-MS	TEM	Quantitative detection of Au NPs in fetal tissue after exposure during early and late pregnancy. Qualitative visualization of Au NPs in fetal brain and liver.
[61]	CD-1 mice	156	Au NPs/ PEG, citrate, or ferritin	13^b^	i.v./ 0, 0.9, or 7.2 mg/kg/ GD5.5–15.5^e^	ICP-MS	TEM, in vivo fluorescence imaging, fluorescence microscopy, and X-ray microscopy	Accumulation of the three Au NP types in extra-embryonic tissue and fetus according to surface composition. Higher Au NP levels during early gestation compared to late gestation.
[62]	Kunming mice	48	CdTe/CdS core/shell QDs/ MPA, SiO_2_, or PEG	1.67 ± 0.29, 2.59 ± 0.43, 3.21 ± 0.32, 4.09 ± 1.02, or 4.20 ± 0.86^b^	i.v./ 0, 0.02, 0.05, 0.086, or 0.125 mg/mouse/ GD21^g^	ICP-OES	In vivo fluorescence imaging	Quantitative Cd detection in mice pups after maternal injection with QDs. Cd accumulation increased with decreasing size and increasing dosage of injected QDs. Qualitative assessment unable to demonstrate intact QDs in fetuses.
[63]	Kunming mice	10	CdSe/CdS/ZnS core/shell/shell QDs/ phospho-lipid micelle	n.d.	i.v./ 0 or 0.81 mg/kg/ 14 days before mating^d^	/	Bright-field light microscopy and fluorescence microscopy	No QD accumulation in the placenta following prenatal IV injection of QDs in mice.
[64]	Kunming mice	10	CdSe/ZnS core/shell QDs	13^b^	i.v./ 0 or 12.5 nmol/mouse/ GD13-GD18^d^	ICP-MS	/	Significant elevations in placental Cd levels for pregnant mice exposed to QDs.
[65]	Kunming mice	20	CdSe and CdSe/ZnS QDs	n.d.	i.v./ 0 or 0.1 nmol/mouse/ GD16–17^d^	ICP-MS	/	Cd detected in the placenta after exposure to different types of QDs. But no significant difference in fetal Cd levels for the exposed group compared to the control group.
[66]	CD-1 mice	15 to 63	CdO NPs	11.0 ± 0.1 or 15.3 ± 0.1^c^	nose-only inhalation/ 0, 0.1 mg/m^3^ for 1.25 h every other day or 0.23 mg/m^3^ for 2.5 h/day/ GD4.5–16.5^e^	AAS, ICP-MS	/	Cd accumulation in mouse uterus and placenta, as well as other maternal organs, in an associated way with inhaled CdO NPs. CdO NPs were undetectable in fetuses.
[67]	BALB/c mice	56	CeO_2_ NPs	3–5^b^	i.v./ 0 or 5 mg/kg/ GD5–7^f^	ICP-MS	/	CeO_2_ NPs detected in decidual tissue and placentas of IV treated mice during early gestation.
[68]	C57Bl/6 mice	19	Cu NPs	35.6 ± 1.7^c^	whole-body inhalation/ 0 or 3.5 mg/m^3^ for 4 h/day/ GD3–19^f^	ICP-MS	/	No quantitative detection of Cu in the placental nor fetal tissue of exposed mice.
[69]	CD-1 mice	80	Fe_2_O_3_ NPs/PEI or PAA	n.d.	i.p./0 or 10 mg/kg/ GD9 or 9–16^d^	UV-vis spectrophoto-meter	Bright-field light microscopy	Both Fe_2_O_3_ NP types crossed the placenta. Only mice treated with PEI-NPs for eight consecutive doses showed a significant increase in Fe levels in fetal livers and placentae.
[70]	Wistar rats	8	MMSNPs/ [^99m^Tc]	58.9 ± 8.1^b^	i.v./ 0 or 18.5 MBq/mL/ GD11 or 20^g^	Gamma spectroscopy	/	SiO_2_ NPs crossed the placenta of pregnant rats, both during early and late stages of gestation. SiO_2_ NPs reach the fetal bloodstream and bioaccumulate in both embryos and fetuses.
[71]	C57Bl/6 mice	11	MMSNPs/ gadolinium oxide-core and TFP	100–200^b^	i.v./ 0 or 1 mg/mouse/ GD7–9 or 14–15^d^	MRI and ultrasound imaging	/	SiO_2_ NPs observed in embryos of mice following early gestation injections while being excluded from the embryo by the placenta following late gestation injection.
[72]	CD-1 mice	44	Pt NPs	20.9 ± 11.4^a^	oral/ 0, 0.25, 0.5, or 1 mg/kg/ 14 days before mating-PND4^g^	ICP-MS	/	No detection of Pt NPs in pups of mice orally exposed before, during, and after gestation.
[73]	BALB/c mice	n.d.	SiO_2_ and TiO_2_ NPs/ fluorophore	35, 70, 300, or 1000^a^	i.v./ 0 or 0.8 mg/mouse/ GD16 or 16–17^g^	/	In vivo fluorescence imaging, fluorescence microscopy, and TEM	Only smaller SiO_2_ and TiO_2_ NPs found in the placenta, fetal liver, and fetal brain.
[74]	CD-1 mice	70	SiO_2_ NPs/ amine or carboxyl	25, 60, or 115^a^	i.v./ 0 or 0.2 mg/mouse/ GD5.5, 12,5, or 16.5^e^	ICP-OES	/	SiO_2_ NPs administered at different gestational stages reached placenta and fetus. Biodistribution influenced by NP size, surface charge, and gestational stage.
[75]	Wistar rats	30	TiO_2_ NPs	21^b^	oral/ 0 or 200 mg/kg/ GD6–12^d^	/	TEM and SEM/EDX	TiO_2_ NPs bypass the placenta and reached late-term neonatal rat lung tissue.
[76]	CD-1 mice	20	TiO_2_ NPs	6.5^a^	oral/ 0, 25, 50, or 100 mg/kg/ GD0–17^d^	ICP-MS	/	Significantly increased Ti content in placenta and fetus with received TiO_2_ NP dose compared to controls.
[77]	C57Bl/6 mice	45	TiO_2_ NPs	97^c^	Whole-body inhalation/ 0 or 42 mg/m^3^ for 1 h/day/ GD8–18^g^	ICP-MS	/	No quantitative detection of Ti in mice pups following maternal inhalation of nanosized TiO_2_.
[78]	Wistar rats	12	TiO_2_ NPs	10^a^	oral/ 0 or 100 mg/kg/ GD2–21^g^	ICP-MS	/	Ti accumulated in hippocampus of rat offspring after gestational TiO_2_ NP exposure.
[79]	C57Bl/6 mice	15	TiO_2_ NPs	5–6^b^	i.v./ 0, 0.1, or 1 mg/mouse/ GD9^d^	SF-ICP-MS	/	No significant accumulation of Ti in maternal plasma, placenta, fetal liver, and fetal brain for the 3 groups exposed to different concentrations of TiO_2_ NPs.
[80]	CD-1 mice	12	TiO_2_ NPs	25–70^a^	s.c./ 0 or 0.1 mg/mouse/ GD3, 7, 10, and 14^g^	/	FE-SEM/EDX	TiO_2_ NP transfer from pregnant mice into the brain and testis of their offspring.
[81]	SPF mice	20	TiO_2_ NPs	5.5^a^	oral/ 0, 1.25, 2.5, or 5 mg/kg/ prenatal day 7-PND21^g^	ICP-MS	/	Maternal gestational exposure to TiO_2_ NPs enhanced Ti content in offspring’s’ hippocampi.
[82]	Sprague Dawley rats	4	ZnO NPs/ citrate	20^a^	oral/ 0 or 400 mg/kg/ GD5–19^d^	ICP-OES	/	No significant difference in fetal Zn content between control and ZnO NP exposed group.
[83]	Sprague Dawley rats	10	ZnO NPs/ APTES	> 35^a^	i.v./ 0 or 20 mg/kg/ GD6–20^d^	ICP-MS	/	Significantly elevated Zn levels in fetal liver after IV injection of pregnant rats with ZnO NPs.
[84]	Sprague Dawley rats	24	ZnO NPs	< 100^a^	oral/ 0 or 500 mg/kg/ 14 days before mating-day 4 of lactation^g^	ICP-MS	/	Significantly higher levels of Zn in liver and kidneys, but not in blood and brain of rat offspring exposed to ZnO NPs before, during, and after gestation.
[85]	CD-1 mice	40	ZnO NPs	13.2 ± 3.7, 57.1 ± 4.1, or 1900 ± 504^b^	oral/ 0 or 7.2 mg/mouse/ GD1–10 or 7–16^f^	ICP-MS	/	Zn detected in the placentae of mothers exposed to ZnO NPs during early gestation in contrast to mothers exposed to bulk ZnO. Only the smallest ZnO NPs crossed the placenta to reach the fetus.
[86]	CD-1 mice	60	ZrO_2_ NPs	16 ± 4^b^	oral/0, 2.5, 25, or 50 mg/kg/ GD9–11, GD13–15, or GD16–18^f^	ICP-MS	TEM/EDX	Fetal accumulation of ZrO_2_ NPs following oral exposure of pregnant mice during different stages of pregnancy.
**Carbonaceous NPs**								
[87]	Sprague Dawley rats	30	Fullerene/ [^14^C(U)]	26 ± 7^b^	i.v./ 0 or 0.2 mg/kg/ GD11, 15, or 18^g^	Gamma spectroscopy	/	Radioactive signals from C60 NPs detected in placenta and fetuses of exposed pregnant dams. Stronger signal 24 h compared to 8 days post-injection.
[88]	Sprague Dawley rats	8	Fullerene/ [^14^C(U)]	< 10^a^	i.v./ 0 or 0.3 mg/kg/ GD 15^g^	Gamma spectroscopy	/	Radioactive signals detected in the placenta and fetuses of pregnant dams, indicative of transplacental C60 NP transfer.
**Polymeric NPs**								
[89]	Wistar rats	24	PGMA NPs/PEI, fluorophore, and magnetite core	n.d.	i.v./ 0 or 0.5 mg/rat/ GD10 or 20^f^	MRI and fluorescence microscopy	Ex vivo fluorescence imaging and confocal microscopy	Both PGMA NP types detected in the rat conceptus during early gestation. Greater accumulation of cationic NPs within the chorionic plate than anionic NPs.
[27]	FVB/N mice	40	PS NPs/ carboxyl and fluorophore	20, 40, 100, 200, and 500^a^	i.v./ 0.3 mg/mouse/ GD17^g^	HPLC and fluorescence microscopy	/	Placental uptake and transfer of fluorescent PS NPs with diameters up to 500 nm. NPs observed in various organs of fetuses after 4 h of administration to pregnant mice.
[90]	Mice	15	PS NPs/ fluorophore and PEG or carboxyl	50–70^a^	i.v./ 0 or 0.00231 mg/kg/ GD10–15^g^	/	Confocal microscopy	Both PS NP types found in placenta but not in embryonic tissues.
**Ambient (ultra)fine particles**								
[91]	New-Zealand white rabbits	8	DEP	69^c^	nose-only inhalation/ 0 or 1 mg/m^3^ for 2 h/day, 5 days/week/ GD3–27^g^	/	TEM	NP-like structures observed in olfactory tissues of fetuses from DEP exposed mothers.
[92]	New-Zealand white rabbits	n.d.	DEP	69^c^	nose-only inhalation/ 0 or 1 mg/m^3^ for 2 h/day, 5 days/week / GD3–27^g^	/	TEM	NP-like structures observed in placenta, maternal blood space, trophoblasts and fetal blood of exposed rabbits.

Data are shown as mean ± standard deviation, ^a^primary particle size stated by the manufacturer, ^b^primary particle size determined by TEM, ^c^Geometric size determined by SMPS, ^d^GD0 = sperm positive/vaginal plug positive, ^e^GD 0.5 = sperm positive/vaginal plug positive, ^f^GD1 = sperm positive/vaginal plug positive, ^g^GD0 not defined

Abbreviations - *AAS* atomic absorption spectrometry, *Ag* silver, *Al*_*2*_*O*_*3*_ aluminum oxide or alumina, *AMG* autometallography, *APTES* 3-aminopropyl triethoxydsilane, *Au* gold, *CdO* cadmium oxide, *CdS* cadmium sulfide, *CdTe* cadmium telluride, *CdSe* cadmium selenide, *d* diameter, *DEP* diesel exhaust particles, *DLS* dynamic light scattering, *DMSA* dimercaptosuccinic acid, *EDX* energy-dispersive X-ray spectroscopy, *Fe*_*2*_*O*_*3*_ iron oxide, *FE-SEM/EDX* field emission-type scanning electron microscopy/energy-dispersive X-ray spectroscopy, *GD* gestation day, *HPLC* high-performance liquid chromatography, *ICP-MS* inductively coupled plasma-mass spectrometry, *ICP-OES* inductively coupled plasma-optical emission spectrometry, *i.p.* intraperitoneal, *i.v.* intravenous, *MBq* megabecquerel, *MMSNPs* magnetic mesoporous silica nanoparticles, *MPA* 3-mercaptopropionic acid, *MRI* magnetic resonance imaging, *n.d.* not defined, *NP* nanoparticle, *NTA* nanoparticle tracking analysis, *PAA* poly(acrylic acid), *PEG* poly(ethylene glycol), *PEI* poly(ethyleneimine), *PGMA* poly(glycidyl methacrylate), *PND* postnatal day, *PS* polystyrene, *PVP* polyvinylpyrrolidone, *QD* quantum dot, *s.c.* subcutaneous, *SF-ICP-MS* sector field inductively coupled plasma-mass spectrometry, *SiO*_*2*_ silicon dioxide or silica, *SMPS* scanning mobility particle sizer, *S-TPP* sulfonated triphenylphosphine, *TEM* transmission electron microscopy, *TFP* trifluoropropyl, *TiO*_*2*_ titanium dioxide, *ZnO* zinc oxide, *ZnS* zinc sulfide, *ZrO*_*2*_ zirconium dioxide

### In vivo maternal-fetal particle transfer in humans

Only 2 studies examined the maternal-fetal transfer of (ultra)fine particles and NPs under real-life exposure conditions [1, 93] (Table 4). Bové et al. performed a study on a subset of term placentae from 20 healthy, non-smoking mother-newborn pairs enrolled within the Belgian ENVIR*ON*AGE (ENVIRonmental influence *ON* early AGEing) birth cohort [94]. Ambient black carbon (BC) particles were found in all screened placentae, and the placental BC load was positively associated with the mothers’ residential BC exposure during pregnancy. The average (SD) placental BC load was 0.95 × 10^4^ (0.66 × 10^4^) and 2.09 × 10^4^ (0.90 × 10^4^) particles per mm^3^ for low- and high-exposed mothers, respectively [1]. Raia-Barjat et al. assessed NP crossing over the human placenta by investigating the NP loading in amniotic fluids collected from 100 pregnant women. A high number of pregnant women with a substantial concentration of the essential trace elements iron (Fe), copper (Cu), and zinc (Zn) in the nano/ion fraction was observed. In contrast, the prevalence of women with a substantial concentration of aluminum (Al), silver (Ag), beryllium (Be), cobalt (Co), chromium (Cr), nickel (Ni), silicon (Si), titanium (Ti), and tungsten (W), was relatively low (i.e.*,* under 20%). Nonetheless, the authors acknowledged that this does not necessarily indicate the presence of NPs since the used technique, ICP-OES, is not able to discriminate NPs from ions [93].

**Table 4:** Basic characteristics of the human study included in the present systematic review investigating the maternal-fetal transfer of ambient (ultra)fine particles

Ref	Sample size	Exposure			Detection technique		Main findings
		Particle	Route	Dose	Quantitative	Qualitative	
[1]	20	BC particles	Real-life exposure	0.6–2.4 μg/m^3 a^	Two-photon fs pulsed laser microscopy	TEM	Ambient BC particles found in all screened placentas and positively associated with the mother’s residential BC exposure during pregnancy.
[93]	100	Metallic NPs	Real-life exposure	n.d.	ICP-OES	SEM/EDX	High prevalence of essential trace elements Cu, Fe, and Zn in the nano/ion fraction observed in amniotic fluid of pregnant women. In contrast, low concentrations and low prevalence of other elements. No conclusions on transplacental NP transfer.

Abbreviations - *BC* black carbon, *Cu* copper, *Fe* iron, *fs* femtosecond, *ICP-OES* inductively coupled plasma-optical emission spectrometry, *m3* cubic meter, *μm* micrometer, *n.d.* not defined, *NPs* nanoparticles, *SEM/EDX* scanning electron microscopy/energy-dispersive X-ray spectroscopy, *TEM* transmission electron microscopy

^a^predominantly inhaled

### Analytical methods to visualize and quantify maternal-fetal particle transfer

A variety of analytical methods were used to quantify (i.e.*,* (semi-)quantitative techniques) and/or visualize (i.e.*,* qualitative techniques) the gestational transfer of particles by their identification in relevant tissues (e.g.*,* placental or fetal tissue). Included studies based their results on (semi-)quantitative detection techniques (*N* = 33), qualitative methods (*N* = 11), or a combination of both (*N* = 29). In total, 26 different techniques were used in the studies discussed in this review, as summarized in Table 5.

**Table 5:** Overview of the commonly employed methods used to qualitatively and quantitatively assess maternal-fetal particle transfer

Ref	Method	(Semi-) Quantitative and/or qualitative assessment	Strengths	Limitations	NPs studied
**Imaging techniques**					
[23, 24, 33, 37, 63, 69, 95]	Bright-field light microscopy [96–99]	NP visualization	Easy, rapid, low cost, non-destructive	Low contrast, staining artifact, no NP sizing	Ag, Au, Fe_2_O_3_, Fe_3_O_4_, SPIONs, and magnetic NPs
[24–27, 32, 33, 89, 90]	Confocal microscopy [96, 97, 100]	NP visualization	High sensitivity, 3D reconstruction (optical sectioning), increased optical resolution (no out-of-focus signals), multiplexing capabilities, non-destructive	Photobleaching, uncoupling or leakage of fluorophores, no NP sizing	PS, PGMA, SPIONs, and SiO_2_ NPs
[61, 62, 73, 89]	Ex vivo*/*in vivo fluorescence imaging [101]	NP visualization	Easy, low cost, non-invasive, multiplexing capabilities, whole-body imaging possible, not sample destructive, real-time	Limited imaging depth (tissue penetration < 1 cm, autofluorescence), photobleaching, uncoupling or leakage of fluorophores, no NP sizing	QDs, Au, PS, SiO_2_, and TiO_2_ NPs
[24–28, 35, 40–42, 61–63, 73, 89]	Fluorescence microscopy [96–99]	NP visualization	Easy, low cost, multiplexing capabilities, non-destructive	Limited (axial) resolution and imaging depth (autofluorescence), photobleaching, uncoupling or leakage of fluorophores, no NP sizing	Au, PGMA, PS, SiO_2_, and TiO_2_ NPs
[38, 46]	Hyperspectral imaging [102, 103]	NP visualization	Easy, multiplexing capabilities, improved SNR (differentiation of NP signal from autofluorescence), high specificity, non-destructive	No NP sizing	Ag and Au NPs
[71, 89]	MRI [96]	NP visualization	High resolution, non-invasive, non-destructive, whole-body imaging, real-time, not limited by tissue depth	Restricted to magnetic NPs, slow image acquisition and long post-processing times, uncoupling of contrast agents, no NP sizing	SiO_2_ and PGMA NPs
[75, 80, 93]	SEM [96, 99]	NP-cell interaction and visualization	High resolution, combination with EDX for elemental analysis, no quenching/bleaching/uncoupling effects	Time-consuming, expensive, destructive, staining and shrinking artifacts, only applicable for electron-dense NPs, no NP sizing, not suitable for living material	TiO_2_ NPs
[1, 23, 24, 27, 29–32, 41, 43, 45, 47, 49, 52, 54, 58, 60, 61, 73, 75, 91, 92]	TEM [96, 104]	Ultrastructural analysis and (subcellular) NP visualization	High resolution, combination with EDX for elemental analysis, no quenching/bleaching/uncoupling effects	Time-consuming, expensive, destructive, staining and shrinking artifacts, only applicable for electron-dense NPs, no NP sizing, not suitable for living material	Ag, Au, BC, DEP, Fe_3_O_4,_ SiO_2_, TiO_2,_ and PS NPs
[1]	Two-photon fs pulsed laser microscopy [105]	NP visualization	High sensitivity and specificity, label-free, non-destructive	No NP sizing	BC particles
[71]	Ultrasound imaging [106]	NP visualization	Low cost, real-time, non-destructive	Sensitive to blood flow and tissue elasticity, uncoupling of contrast agents, no NP sizing	SiO_2_ NPs
[61]	X-ray microscopy [107, 108]	NP visualization	High resolution, high specificity and sensitivity, large penetration depth	Destructive, radiation damage, no NP sizing	Au NPs
**Spectroscopic techniques**					
[33, 44, 48, 49, 59, 66]	AAS [109]	Elemental composition, NP quantification (LoD: high ppb range)	Accurate, fast, easy, high sensitivity and specificity	Time-consuming, expensive, no information on cellular NP localization	SPIONs, Ag, Au, and CdO NPs
[53, 57, 70, 87, 88]	Gamma spectroscopy [96]	Identification and quantification of radioisotope-labeled NPs	High sensitivity and specificity,	Expensive, radioactive labeling, radiation safety requirements, limited spatiotemporal resolution	Fullerene, Ag, Au, and SiO_2_ NPs
[22, 23, 31, 32, 38, 45–47, 50–52, 55, 58, 60, 61, 64–68, 72, 76–78, 81, 83–86]	ICP-MS [110, 111]	Elemental composition, NP quantification (LoD: ppt range)	Rapid, high sensitivity and specificity, little sample preparation (no labeling needed), high sample throughput (all elements 2–6 min)	Chemical interference (e.g.*,* argon from plasma), dissolution of NP, quantification of non-metal-based NPs not possible, no information on cellular NP localization	QDs, Ag, Au, CdO, CeO_2_, Cu, ZnO, and TiO_2_ NPs
[54, 62, 74, 82, 93]	ICP-OES [112, 113]	Elemental composition, NP (cellular internalization) quantification (LoD: low ppb range)	Reproducible, high sensitivity and specificity, no chemical interference, little sample preparation (no labeling needed), high sample throughput (5–30 elements/min)	Spectral interference, dissolution of NP, quantification of non-metal-based NPs not possible, no information on cellular NP localization	QDs, Ag, and SiO_2_ NPs
[33]	MPS [114]	NP quantification	High sensitivity, little sample preparation (no labeling nor purification needed)	Time-consuming, expensive, quantification of non-magnetic NPs not possible	SPIONs
[69]	UV-Vis spectroscopy [115, 116]	NP quantification	Easy, fast	Low sensitivity, no information on cellular NP localization	Fe_2_O_3_ NPs
**Other techniques**					
[36]	AF4 (UV detection) [117, 118]	NP quantification	High resolution, highly reproducible, rapid, size separation possible	Low sensitivity, no information on cellular NP localization	PS NPs
[27]	Flow cytometry [97]	NP (cellular uptake) quantification	Easy, rapid, high sample throughput, multiplexing capabilities, not sample destructive	No information on cellular NP localization, uncoupling or leakage of fluorophores	PS NPs
[27]	HPLC (fluorescence detection) [118]	NP quantification	Rapid, size separation possible	No information on cellular NP localization, uncoupling or leakage of fluorophores	PS NPs

Abbreviations - *AAS* atomic absorption spectrometry, *Ag* silver, *Au* gold, *BC* black carbon, *CdO* cadmium oxide, *CeO*_*2*_ cerium dioxide, *Cu* copper, *DEP* diesel exhaust particles, *EDX* energy-dispersive X-ray spectroscopy, *Fe*_*2*_*O*_*3*_ iron oxide, *Fe*_*3*_*O*_*4*_ iron oxide or magnetite, *fs* femtosecond, *ICP-MS* inductively coupled plasma-mass spectrometry, *ICP-OES* inductively coupled plasma-optical emission spectrometry, *LoD* limit of detection, *MRI* magnetic resonance imaging, *NP* nanoparticle, *PGMA* poly(glycidyl methacrylate), *PS* polystyrene, *QD* quantum dot, *TEM* transmission electron microscopy, *TiO*_*2*_: titanium dioxide, *SEM* scanning electron microscopy, *SiO*_*2*_ silicon dioxide or silica, *SPIONs* superparamagnetic iron oxide nanoparticles, *TEM* transmission electron microscopy, *TiO*_*2*_ titanium dioxide, *UV* ultraviolet, *UV-Vis* ultraviolet-visible, *ZnOs* zinc oxide

#### Qualitative techniques

Qualitative microscopy-based methods employed in the discussed studies included bright-field light, fluorescence, and confocal microscopy as well as transmission and scanning electron microscopy (TEM and SEM), and X-ray microscopy. The most frequently used qualitative technique was TEM (*N* = 22). Analytical tools, such as energy-dispersive X-ray spectroscopy (EDX) or Raman spectroscopy, can be coupled to electron microscopes for additional elemental composition analysis [45, 47, 75, 80, 93]. For example, Takeda et al. used field emission-type SEM (FE-SEM)/EDX to show the transfer of nanosized TiO_2_ from pregnant mice into the brain and testis of their offspring [80]. Autometallography, a histochemical technique based on silver enhancement, is also used to allow light and electron microscopic visualization of metallic NPs in biological tissues and cells (*N* = 4). Using TEM and silver enhancement, 10 and 15 nm Au PEGylated NPs could be visualized in the trophoblastic cell layer of the placenta after 6 h of re-circulating perfusion, yet no particles were quantified in the fetal circulation [23].

#### Quantitative techniques

One of the included human studies on maternal-fetal particle transfer employed a novel detection technique based on the white-light generation by carbonaceous particles under near-infrared femtosecond pulsed illumination [1, 105]. This label-free confocal microscopic technique showed the presence of BC particles originating from ambient exposure at the fetal side of the human placenta [1]. Label-free detection of NPs in cells and tissues is not straightforward and, therefore, NPs are often labeled using fluorophores, radiolabels, or contrast agents. For quantitative visualization of non-fluorescent NPs in biological systems, fluorescent labeling (e.g.*,* using fluorescein isothiocyanate [71] or rhodamine [24, 25]) can be employed followed by fluorescence detection methods. Melnik et al. used the radioactive silver isotope ^110m^Ag to label and track PVP-stabilized 34.9 nm Ag NPs with gamma spectrometry, showing their accumulation in placental and fetal tissue following oral administration to pregnant mice on GD20 [53]. Moreover, Sweeney et al. functionalized mesoporous SiO_2_ NPs with gadolinium oxide (i.e.*,* magnetic resonance imaging (MRI) contrast agent) and trifluoropropyl groups (i.e.*,* ultrasound contrast agent) to allow multimodal imaging of silica NPs in pregnant mice. A time-dependent transfer was observed as SiO_2_ NPs were only detected in the pups following maternal i.v. injection during early gestation (GD9) and not late gestation (GD14) [71]. Other quantitative analysis methods are mainly based on elemental analysis. Inductively coupled plasma (ICP) techniques, including ICP-optical emission spectroscopy (OES) and ICP-mass spectrometry (MS), are powerful tools for the detection and analysis of trace elements in homogenized tissue samples. ICP-MS, or a variant of it (i.e.*,* single particle, sector field, and laser ablation ICP-MS), was the most frequently used quantitative technique (*N* = 29) among the included studies. In addition, 6 of the included studies used atomic absorption spectrometry (AAS) as elemental analysis tool for detection of metallic NPs in maternal-fetal tissue [33, 44, 48, 49, 59, 66].

#### Combination of qualitative and quantitative techniques

In total, 29 included studies combined quantitative and qualitative methods to gain complementary insights on the maternal-fetal transfer of NPs. For example, Ho et al. combined findings from quantitative (i.e.*,* magnetic resonance imaging and fluorometry) and qualitative assessments (i.e.*,* fluorescence and confocal imaging) to determine the biodistribution of cationic and anionic multimodal (i.e.*,* fluorescent and paramagnetic) polymeric PGMA NPs in a pregnant rat model at different stages of gestation. While the quantitative methods were unable to unambiguously determine tissue uptake, confocal microscopy confirmed a differential charge-based accumulation of the NPs in the rat placenta [89]. The time-dependent transfer of SPIONs with a differential surface charge through a BeWo/pericyte co-culture was measured by magnetic particle spectroscopy and confirmed by AAS. As previously observed, neutral and negatively charged iron oxide NPs were able to pass the cell layer, whereas positively charged NPs primarily interact with the BeWo cells [33].

## Discussion

### Placenta models used to study maternal-fetal NP translocation

As visualized in Fig. 2a, only 2 included studies examined the transplacental passage of (ultra)fine particles or NPs in a human population under real-life exposure conditions [1, 93]. The majority of the cited studies investigated maternal-fetal particle transfer and distribution in animal models, hence, in biological systems under controlled conditions. Mainly rodents (i.e.*,* mice and rats) were used as animal model (Fig. 3a). Both rodents and rabbits are easy to breed and handle and their small size facilitates large scale/high throughput studies, making them cost-efficient models [119, 120]. However, placentation differs between species [121]. To compare data on transplacental NP transfer, it is necessary to understand both the physiological and anatomical differences between humans and the employed animal models [122]. Humans, rodents, and lagomorphs (rabbits) all share a discoid, hemochorial placenta, denoting that maternal blood comes in direct contact with fetal trophoblastic tissue. More specifically, hemomonochorial (human at term), hemodichorial (rabbit and human in their first trimester), and hemotrichorial (rat and mouse) placentae, with one, two, and three trophoblastic epithelial layers separating maternal and fetal blood, respectively [121]. Rodents are the most widely used animal model in developmental toxicology, yet there are structural differences between rodent and human placentae. Rodents (i) reach their definitive placental structure in a later stage, (ii) have less invasive trophoblast cells, and (iii) have a labyrinthine as opposed to a villous organization in human placentae [123, 124]. Rabbit placentae also have a labyrinthine structure, yet they are hemochorial with two trophoblast layers, a syncytium and a cytotrophoblast layer, which more closely resembles the human placenta. Moreover, critical exposure windows can be defined more precisely in rabbits as ovulation is induced by mating, resulting in the exact timing of fertilization and pregnancy stages. Rabbit placentae appear to resemble more the human placentae than that of rodents. Therefore, rabbits are considered the preferable animal model to study gestational particle exposure [120, 125]. Keeping in mind that the placenta is the most species-specific mammalian organ, various human model systems have been developed to mimic the in vivo situation in pregnant women as closely as possible.

Ex vivo perfusion of human term placentae is used as a surrogate to study the transplacental transport of NPs. It is considered as the “gold standard” among currently available translocation models as it preserves the structural complexity of a full-term placenta and resembles its dynamic environment, enabling to study ex vivo transplacental NP passage without harming the mother and/or fetus [126]. Nonetheless, perfusion studies (i) predominantly use full-term placentae and, hence, do not allow to estimate NP transfer during earlier and more vulnerable stages of pregnancy, (ii) are limited to a few hours (4–8 h) of perfusion due to tissue degradation, which is insufficient to observe chronic effects, (iii) have a low success rate of perfusion (i.e.*,* 30%), and (iv) are time-consuming [126, 127].

To overcome the aforementioned limitations, in vitro models using human cell cultures (primary cytotrophoblasts or choriocarcinoma cell lines) are attractive as they allow high-throughput testing of transplacental transfer of various NPs. However, models like the commonly employed BeWo b30 Transwell model also have their limitations [128]. In vitro placental transfer models do not fully resemble the physiological structure of the in vivo placenta as they lack anatomical integrity and blood flow. Moreover, monolayers form a simplified placental barrier. Co-cultures, on the other hand, attempt to more closely mimic the complex in vivo placenta that constitutes multiple cell layers (e.g.*,* trophoblast and endothelial cell layer) across which particles have to be transported and where cell-cell interactions among various cell types can happen [129, 130]. In this regard, Muoth et al. used a 3D co-culture microtissue model to more closely resemble the human placental structure, and to study Au NP uptake and penetration in an organotypic environment. Higher uptake and deeper penetration were observed for the smaller carboxylated Au NPs in comparison to the larger PEGylated Au NPs [32]. Interestingly, a lower degree of NP transport was observed across co-culture placental models compared to monolayer cultures. For instance, Cartwright et al. exposed a BeWo b30 monolayer to PS NPs with sizes up to 100 nm and was able to detect those particles in the basal compartment after 24 h of exposure [26]. Correspondingly, Aengenheister et al. exposed a BeWo b30/HPEC-A2 co-culture to PS NPs with particle sizes up to 70 nm under similar conditions and found only low amounts of 49 nm PS NPs and no 70 nm PS NPs in the basal compartment after 24 h of exposure [35]. Additionally, Au NPs (±14 nm) were able to transport across BeWo b30 and HVMF monolayers, while they barely passed the co-culture of both cell types [32]. Nonetheless, most in vitro models lack a physiological microenvironment as they are exposed to NPs under static conditions.

Although a variety of placental models are currently available, the preconditions of each model should be taken into consideration depending on the research objective. High-throughput in vitro transfer models are useful to pre-screen a variety of NPs and to provide mechanistic insights, yet, further improvements (e.g.*,* microfluidic approaches to develop a dynamic model) are needed to enhance their predictive value. Ex vivo placental perfusion studies provide transfer data with high in vivo relevance, at least for term pregnancy. On the other hand, exposure of pregnant animal models can provide important insights on the biodistribution of NPs in a living organism, including potential translocation to the fetus. Nevertheless, data extrapolation from animals to humans remains challenging as the placenta is the most species-specific mammalian organ. Despite technical challenges and ethical constraints, term human placentae form the ideal model to study transplacental NP transfer.

### Evidence of maternal-fetal NPs translocation

The majority of the cited studies observed transplacental transfer of (ultra)fine particles and NPs (Fig. 2c). This supports the finding that the placenta is not an impenetrable barrier, as already confirmed for other xenobiotics such as drugs and alcohol [131]. In animal models, transplacental passage has been reported for Ag NPs [47–54], Al_2_O_3_ NPs [44], Au NPs [57, 59–61], CeO_2_ NPs [67], DEP [91], QDs [62], SiO_2_ NPs [70, 71, 73, 74], TiO_2_ NPs [73, 75, 76, 78, 80, 81], ZnO NPs [83–85], ZrO_2_ NPs [86], Fe_2_O_3_ NPs [69], C60 fullerenes [87], PGMA NPs [89], and PS NPs [27]. Among these particles, only Ag NPs [37], Au NPs [31, 38], and PS NPs [40–43] showed transplacental transfer in an ex vivo perfusion model, whereas SiO_2_ NPs [25] and TiO_2_ NPs [34] were retained in the placental tissue. Similarly, Ag NPs [22], Au NPs [31, 32], iron oxide NPs [24, 33], SiO_2_ NPs [24], and PS NPs [35] were shown to cross the in vitro placental barrier. Solely 22 studies based their findings on visual confirmation of particle presence or a significant difference in NP content from the control group in the fetal compartment/tissue. Moreover, 22 studies substantiated their evidence for maternal-fetal NP translocation by determining the limit of detection, i.e.*,* values exceeding the empirically defined size and/or concentration limits. Nonetheless, other studies reported contradictory results and report the absence of NP placental transfer or fetal uptake of Au NPs [56], Cu NPs [68], Pt NPs [72], TiO_2_ NPs [77, 79], QDs [63], and ZnO NPs [82]. Yet, this may be due to inadequate size and concentration detection limits of the employed techniques, as discussed in the section “advantages and disadvantages of methods used to assess maternal-fetal NP translocation”.

### Factors that influence maternal-fetal translocation

Various studies showed the influence of different factors on maternal-fetal particle translocation, including particle size, particle material, dose, particle dissolution, and surface composition, as well as NP administration route and the gestational stage of the study model.

First, the effect of particle size on transplacental transfer was addressed by 6 included studies [27, 55, 57, 62, 73, 85]. For instance, Yamashita et al. i.v. administered 70, 300, or 1000 nm SiO_2_ particles to pregnant mice and concluded that only the 70 nm particles could reach the placentae, fetal liver, and fetal brain tissue [73]. Similar size-dependent transplacental transfer was observed for PS NPs in the ex vivo placental perfusion [40, 42, 43] and in vitro [26, 27, 35] studies. Different size-dependent mechanisms of placental NP exchange have been proposed, including passive and facilitated diffusion via transtrophoblastic channels (i.e.*,* canaliculi) and active transport (e.g.*,* receptor-mediated endocytosis), yet mechanistic insights on transplacental NP transfer remain scarce [39]. Despite the use of various placental transporter inhibitors, no major influence on the translocation of 50 nm PS NPs in the in vitro BeWo transfer model was observed, which indicates preferential NP translocation by passive diffusion [28]. In contrast, bidirectional ex vivo placental perfusion studies showed an increased fetal to maternal transfer of 50 nm PS NPs suggesting an active, energy-dependent transplacental transport mechanism for PS NPs [41]. Additionally, upregulation of clathrin- and caveolin-mediated endocytosis was observed following i.v. administration of 20 and 50 nm Au NPs [55] and oral administration of 16 nm ZrO_2_ [86] in pregnant mice. Noteworthy, the particle cut-off size, above which no transplacental particle translocation is observed, appears to be influenced by the particle material. Aengenheister et al. showed the in vitro transplacental transfer of 50 nm PS [35] but not 4–8 nm TiO_2_ NPs [34] across a BeWo/HPEC co-culture. The study also showed that the 4–8 nm TiO_2_ NPs were not able to cross the placenta in an ex vivo perfusion model [34], whereas PS NPs up to 240 nm have been shown to reach the fetal circuit [43] in a similar perfusion study. In agreement, in pregnant mice, 5 nm TiO_2_ did not cross the placenta [79], while PS NPs up to 500 nm could be observed in various organs of fetuses [27]. Moreover, Kloet et al. observed a difference in translocation behavior of PS NPs with a similar size and surface charge but acquired from different manufactures [28]. This reflects the difficulty to compare results between, and even within, studies due to variations in NP characteristics on top of differences in the employed detection technique.

Second, numerous studies reported that the surface composition of the particles has a tremendous influence on their in vivo translocation [22, 24, 31–34, 37, 41, 42, 61, 62, 69, 74, 89, 95, 96]. Accordingly, Yang et al. exploited three types of surface modifications to assess the effect of surface functionality on maternal-placental-fetal biodistribution of 13 nm Au NPs in fetuses of mice; coating with (i) endogenous proteins (i.e.*,* ferritin) for optimal biocompatibility, (ii) stealth groups (i.e.*,* PEG polymer chains) to increase circulation time by avoiding recognition and phagocytosis by the mononuclear phagocytic system, and by reducing NP-cell and NP-protein interactions, and (iii) stabilizing anionic material (i.e.*,* citrate) to explore the effects of negative charge on placental and fetal distribution. Substantially less uptake of 13 nm citrate-capped Au NPs in fetal tissues has been found compared to ferritin-modified or PEGylated Au NPs with an identical size [61]. A similar effect of surface charge on the transplacental crossing of metal oxide NPs was demonstrated by coating 14 nm Fe_2_O_3_ NPs with either negatively charged PAA-groups or positively charged PEI-groups. Pregnant mice intraperitoneally exposed to 10 mg/kg Fe_2_O_3_-PEI for eight consecutive days (i.e.*,* GD9 to 16) had significantly increased iron levels in their placentae and the livers of their offspring compared to mice exposed to Fe_2_O_3_-PAA [69]. In general, an increased transplacental transfer for PEGylated NPs was observed among the included studies, while carboxylated NPs were mainly retained inside placental tissue [31, 37, 41, 42]. In accordance, Aengenheister et al. showed that both carboxylated and PEGylated Au NPs crossed the placental co-culture in low amounts. Despite the higher cellular uptake of carboxylated Au NPs, increased translocation was observed for PEGylated Au NPs. In contrast, only PEGylated particles reached the fetal circulation in the dynamic ex vivo placental perfusion model [31]*.* Possible explanations given by the authors for the absence of carboxylated particles in the fetal circulation were: (i) the agglomeration behavior of carboxylated Au NPs, and (ii) the non-specific adherence of carboxylated Au NPs to the perfusion system, both markedly reducing the cellular available dose. In all cases, the carboxylated particles were larger than the PEGylated NPs, which can partially explain the reduced transfer.

Third, another factor with considerable influence on maternal-fetal NP transfer is particle dose as discussed in 9 included studies [24, 45, 48, 62, 66, 76, 79, 81, 86]. By increasing the dosage of i.v. injected 3-mercaptopropionic acid (MPA)-coated QDs in pregnant mice, Chu et al. observed a corresponding increase in Cd concentration in the pups [62]. Similar results were observed for TiO_2_ NPs [76, 81] and ZrO_2_ NPs [86] in a pregnant mouse model. However, a lack of dose-response was observed for CdO [66] NPs and Ag NPs [45, 48], possibly because the tissue saturation limit was not reached by the administered NPs, as suggested by Austin et al. [45, 48]. In general, NP uptake and translocation do not only depend on their physicochemical properties, surface modification, and particle concentration but also on particle dissolution. The latter is an important property to consider because it alters the particle presence. Accordingly, translocation may be observed which cannot only be attributed to intact NPs (e.g.*,* Ag NPs, CdO NPs, and ZnO NPs), but also released ions, precipitates, or a combination. NP dissolution has been reported by 7 [22, 37, 45, 47, 48, 66, 85] of the included studies yet only 2 distinguished between the translocation and uptake of actual particles or dissolved Ag in an in vitro placental barrier model [22] and ex vivo placental perfusion model [37]. Both studies showed a favorable transplacental transport of ionic over particulate Ag and highlighted the need to consider the uptake of Ag ions and/or dissolution of Ag NPs in the cellular barrier or tissue followed by re-precipitation to Ag NPs in the basolateral compartment or fetal circulation, respectively. On the other hand, Wang et al. showed that the integrity of ZrO_2_ NPs was not altered upon encountering biological barriers in a pregnant mouse model as the ionic Zr content was fewer than 1% after incubation of ZrO_2_ NPs with water or artificial gastric fluid for 5 h [86].

Fourth, the administration route in in vivo animal studies is an important factor influencing particle translocation. As depicted in Fig. 3b, inhalation exposure (*N* = 6) remains fairly understudied in gestational translocation studies despite being the main entry route in environmental and occupational settings. Among the included studies, i.v. administration (*N* = 27) is mainly used as route of exposure. i.v. NP injection allows to control the systemically available dose as NPs do not have to cross primary biological barriers (e.g.*,* lung or intestinal epithelium), which would result in limited transfer across the placenta and thus unfeasible detection limits that restrain the visualization and quantification of NPs in embryonic/fetal tissue [132]. However, the data from i.v. route studies cannot directly be extrapolated to the real-life scenario of inhalation exposure due to differences in the systemic distribution pattern [133]. For example, the differences in the overall distribution patterns might be determined by the different protein coronae that arise when particles come into contact with bronchoalveolar fluid compared to plasma [134–136].

Fifth, the previously highlighted interspecies differences are further complicated by dissimilarities in placental anatomy and physiology between the various stages of pregnancy, making it necessary to focus on a critical exposure period in animals that reflects an equivalent stage in human development [137]. Alignment of rodent and lagomorph reproductive timelines with that of humans is based on Theiler (mouse) [138], Witschi (rat) [139], Edwards (rabbit) [140], and Carnegie (human) [141] stages of development. The included studies employed various gestational windows of NP exposure ranging from exposure during early or late gestation [60, 67, 70, 71, 74, 85, 89] to continuous exposure during the whole pregnancy [1, 52, 68, 72, 76, 81, 92, 93] to assess the time-dependence of transplacental particle transfer. In this regard, polymeric NPs accumulated in different compartments of the rat conceptus during early (GD10), but not late (GD20) gestation, consistent with the lack of a developed placenta during early gestation [89]. Similarly, Wang et al. reported a higher translocation of ZrO_2_ NPs to the mouse placenta, fetal brain, and fetal liver following oral exposure during early stages of pregnancy (i.e.*,* GD9–11 and GD13–15) compared to NP administration later in gestation (i.e.*,* GD16–18) [86]. On the other hand, Pietroiusti et al. detected 25 and 60 nm SiO_2_ NPs in the mouse conceptus after i.v. administration at GD5.5 and GD12.5. At later stages (GD16.5), when the placenta acquired higher permeability, larger 115 nm SiO_2_ NPs were able to reach the mouse conceptus [74].

### Methods used to assess maternal-fetal NP translocation

Studies on gestational NP biodistribution demand highly sensitive visualization and quantification methods because the accurate detection of the fetoplacental accumulation of nanomaterials at very low levels is required. The uptake of particulates into the systemic circulation is limited and often amounts less than a few percent of the total administered dose [142]. For example, Hesler et al. showed a lack of transport of 50 nm carboxylated PS NPs across a placental co-culture using an asymmetrical flow field-flow fractionation (AF4) system. Still, they recognize that it cannot be excluded that a small number of particles, below the detection limit of the AF4 system, could have been translocated [36]. This detection limit is possibly confirmed by other studies that were able to show a size-dependent translocation of similar PS NPs in mice [27] as well as in an ex vivo placental perfusion model [41, 42].

As summarized in Table 5, a multitude of imaging approaches was used to visualize NPs in an in vitro*,* ex vivo*,* and in vivo context. Some included studies investigated maternal-fetal NP distribution at the whole-body level by using magnetic resonance imaging [71, 89], in vivo/ex vivo fluorescence imaging [61, 62, 73, 89], or radiolabeling techniques [53, 57, 70, 87]; whereas other reports focused on the subcellular localization of NPs by exploiting transmission [23, 24, 27, 29–32, 41, 43, 45, 47, 49, 52, 54, 57, 58, 60, 61, 73, 75, 91, 92] or scanning electron microscopy [75, 80, 93]. Fluorescence microscopy is generally the method-of-choice to monitor the cellular and tissue-level distribution of fluorescent NPs [143]. However, visualization of fluorescent NPs, even with high-resolution confocal microscopy, is limited because of their sizes (typically being between 1 and 100 nm), which is below the Abbe’s diffraction limit of ~ 250 nm. An additional consideration when using fluorescent NPs is dye leakage [144]. Stable incorporation of dyes within the NPs is a prerequisite as the fluorescence from leaked dyes may cause wrong interpretation of NP biodistribution [145]. Accordingly, the in vitro stability of the fluorescent dye in different solutions, including perfusion medium [42, 43], and phosphate buffered saline [73], was measured in some of the included studies to exclude false-positive results. Moreover, the detection of fluorescent NPs may be hindered by the autofluorescence of the biological samples, which hampers the visualization of small aggregates [143]. Kenesei et al. employed spectral imaging fluorescence microscopy to distinguish fluorescence, originating from the i.v. injected carboxylated or PEGylated PS NPs, from tissue autofluorescence in mice. The study revealed no placental penetration but retention of PS NPs by the reticuloendothelial system regardless of surface functionalization [90]. In general, fluorescence microscopic techniques are hampered by the risk of sample photobleaching combined with limited particle size detection and the necessity to fluorescently couple the NP under study. The latter is facilitated in the case of (i) QDs [62–65], which possess inherent fluorescence, or (ii) carbonaceous particles, which generate white-light under femtosecond pulsed laser illumination [1, 105]. Such label-free detection methods allow the direct analysis of maternal-fetal NP uptake. Chu et al. used fluorescence imaging to investigate whether intact QDs can transfer across the rodent placenta to reach the fetus. Penetration of the placenta by intact QDs was hard to demonstrate using in vivo fluorescence imaging, possibly due to a low fetal concentration combined with a reduction in fluorescence intensity due to background tissue-endogenous fluorescence (i.e.*,* autofluorescence). Nonetheless, increased Cd levels, originating from QDs, were measured in mouse fetuses and pups using ICP-OES [62]. Another study indicated that the Cd ions originating from CdO NPs, instead of the particles themselves, were able to translocate across the placenta of a pregnant mice model [66]. In agreement, favorable transport of ionic silver over pristine Ag NPs was observed across the in vitro placental cell layer and in the ex vivo placental perfusion model [22, 37]. High concentrations of dissolved ions may overestimate the concentration of materials present in nanoparticle form when using chemical analysis-based methods (e.g.*,* ICP-OES and ICP-MS) that measure the total elemental concentration in a sample [146]. In contrast, single-particle ICP-MS (spICP-MS) allows determination of the elemental composition of single particles and provides information on the NP size distribution. Furthermore, spICP-MS can distinguish between dissolved and nanoparticulate forms of a certain material [147]. In this regard, Abdelkhaliq et al. showed the transport of ionic silver and Ag NPs across an in vitro BeWo b30 model using ICP-MS and spICP-MS, respectively [22]. In addition, separation techniques are often used to remove target particles from interfering matrix components to meet the quantification limits of the detection methods. Accordingly, chromatography-based techniques such as field-flow fractionation and high-performance liquid chromatography (HPLC) are used for high-resolution sizing and separation of a wide range of diverse particles [148, 149]. Coupling of the chromatographic instruments with other spectroscopic techniques, such as light scattering or light absorption methods, can be used to enhance the characterization power as it allows the physicochemical characterization and elemental analysis of the size-separated particles [150]. In this regard, Hesler et al. used an AF4-UV method for size analysis and quantification of carboxylated PS NPs across an in vitro placental co-culture [36].

To summarize, quantification and reliable detection of NPs in maternal-fetal tissue remain one of the main challenges in transplacental transfer studies, especially under realistic exposure conditions where transfer rates are expected to be lower. Quantification of maternal-fetal NP uptake is often achieved at the cost of spatial resolution, whereas intracellular NP localization is often qualitatively defined. Hence, techniques are best combined to gain complementary and profound insights in transplacental particle transfer. Moreover, progress should be made towards standardization and validation of methods used to detect (ultra)fine particles and NPs with high specificity to ensure reliable detection of the particles of interest. Such implementation of standardized protocols would facilitate the comparison between different studies on particle exposure and the assessment of associated health and safety risks.

## Conclusion and future directions

Exposure to (ultra)fine particles and NPs in daily life is unavoidable, and this is no different for pregnant women. This systematic review summarizes the evidence that particles can bypass the placenta as observed in (i) in vitro monolayers and co-cultures of different placental cells (e.g.*,* trophoblasts, fibroblastic cells, and endothelial cells), (ii) ex vivo placental perfusion models, and (iii) in vivo rodent and rabbit models and humans. Almost all types of particles tested, ambient or engineered, seemed to be able to reach and/or cross the maternal-fetal barrier, even if merely in trace amounts. Transplacental particle transport is affected by the particle size, particle material, dose, particle dissolution, and surface modification, as well as the NP administration route and gestational stage of the employed model. Results on placental NP transfer must be interpreted with care seeing differences in the species origin (i.e.*,* rodent, rabbit, or human) and complexity (i.e.*,* in vitro*,* ex vivo, or in vivo) of the applied model as well as in particle properties and routes of NP exposure/administration. To obtain substantial and complementary results on developmental toxicity following prenatal exposure, it will be essential to test realistic doses (extrapolated from population-based studies) and exposure routes. The number of studies on transplacental NP transfer remains limited. More studies on the topic are paramount and in particular on inhalation exposure since this is the primary route of environmental and occupational exposure and largely understudied.

Moreover, to date, little is known about the kinetics and bioavailability of NPs as no simple nor standardized method for NP detection and/or quantification in biological settings is available. This urges the need to come up with standardized protocols and to develop state-of-the-art methods to accurately detect and quantify the fetoplacental accumulation of low particle levels in tissues, such as in fetal samples.

To conclude, further research on particle uptake, accumulation, and translocation at the placenta is indispensable to predict potential fetal exposure and adverse health effects during fetal development and later in life.

## Supplementary Information

**Additional file 1.** Protocol.

**Additional file 2.** Review methods description.

## Data Availability

Not applicable.

## Abbreviations

^110m^Ag: Silver-110 m radioisotope
^14^C: Carbon-14 or radiocarbon
3A-sub-E: Human SV40 transformed 3A-Sub-E trophoblast cell line
3D: Three-dimensional
AAS: Atomic absorption spectrometry
AF4: Asymmetrical flow field-flow fractionation system
Al: Aluminum
Al_2_O_3_: Aluminum oxide or alumina
Ag: Silver
Ag_2_S: Silver sulfide
AMG: Autometallography
APTES: 3-aminopropyl triethoxydsilane
Au: Gold
Be: Beryllium
BC: Black carbon
BeWo (b30): Human placental choriocarcinoma cell line
C60: Fullerene
Cd: Cadmium
CdO: Cadmium oxide
CdS: Cadmium sulfide
CdTe: Cadmium telluride
CdSe: Cadmium selenide
CeO_2_: Cerium dioxide
CMX: Carboxymethyl dextran
Co: Cobalt
CO: Carbon monoxide
COOH: Carboxyl
COONa: Carboxyl
Cr: Chromium
Cu: Copper
DEP: Diesel exhaust particles
EDX: Energy-dispersive X-ray spectroscopy
ENVIR*ON*AGE: ENVIRonmental influence *ON* early AGEing
Fe_2_O_3_: Iron oxide
Fe_3_O_4_: Iron oxide or magnetite
FE-SEM/EDX: Field emission-type scanning electron microscopy/energy-dispersive X-ray spectroscopy
fs: Femtosecond
GD: Gestational day
GIT: Gastrointestinal tract
h: Hours
hPC-PL: Primary human placental pericytes
HPEC-A2: SV40-transformed microvascular human placental venous endothelial cells
HPLC: High-performance liquid chromatography
HVMF: Human villous mesenchymal fibroblasts
ICP: Inductively coupled plasma
ICP-MS: Inductively coupled plasma-mass spectrometry
ICP-OES: Inductively coupled plasma-optical emission spectrometry
i.p.: Intraperitoneal
i.v.: Intravenous
kg: Kilogram
LA-ICP-MS: Laser ablation-inductively coupled plasma-mass spectrometry
m^3^: Cubic meter
MBq: Megabecquerel
MeSH: Medical subject headings
mg: Milligram
mL: Milliliter
MMSNPs: Magnetic mesoporous silica nanoparticles
MPA: 3-mercaptopropionic acid
MPS: Magnetic particle spectroscopy
MRI: Magnetic resonance imaging
MS: Mass spectrometry
μg: Microgram
μm: Micrometer
Na: Sodium
n.d.: Not defined
ng: Nanogram
Ni: Nickel
nm: Nanometer
nmol: Nanomolar
NO_2_: Nitrogen dioxide
NP: Nanoparticle
O_3_: Ozone
OES: Optical emission spectroscopy
PAA: Poly(acrylic acid)
PEG: Poly(ethylene glycol)
PEI: Poly(ethyleneimine)
PGMA: Poly(glycidyl methacrylate)
PM_2.5_: Particulate matter smaller than 2.5 μm in diameter
PND: Postnatal day
PRISMA: Preferred Reporting Items for Systematic reviews and Meta-Analyses
PS: Polystyrene
Pt: Platinum
PVP: Polyvinylpyrrolidone
QD: Quantum dot
s.c.: Subcutaneous
SD: Standard deviation
SEM: Scanning electron microscopy
SF-ICP-MS: Sector field inductively coupled plasma-mass spectrometry
Si: Silicon
SiO_2_: Silicon dioxide or silica
SMPS: Scanning mobility particle sizer
SO_2_: Sulfur dioxide
SPIONs: Superparamagnetic iron oxide nanoparticles
S-TPP: Sulfonated triphenylphosphine
TEM: Transmission electron microscopy
TFP: Trifluoropropyl
Ti: Titanium
TiO_2_: Titanium dioxide
UV: Ultraviolet
UV-Vis: Ultraviolet-visible
W: Tungsten
ZnO: Zinc oxide
ZnS: Zinc sulfide
ZrO_2_: Zirconium dioxide
