# Supplementary material for: Translocation of (ultra)fine particles and nanoparticles across the placenta; a systematic review on the evidence of in vitro, ex vivo, and in vivo studies
Source: Part Fibre Toxicol. 2020 Nov 2;17:56. doi: 10.1186/s12989-020-00386-8 (PMC7607677; doi:10.1186/s12989-020-00386-8)
Supplement: Supplementary file 1 — Additional file 1. Protocol. [file 12989_2020_386_MOESM1_ESM.pdf]

Systematic review

This record cannot be edited because it is being assessed by the editorial team

1. * Review title.

Give the working title of the review, for example the one used for obtaining funding. Ideally the title should state succinctly the interventions or exposures being reviewed and the associated health or social problems. Where appropriate, the title should use the PI(E)COS structure to contain information on the Participants, Intervention (or Exposure) and Comparison groups, the Outcomes to be measured and Study designs to be included.

Translocation of nanoparticles across the placental barrier; a systematic review protocol on the evidence of in vitro, ex vivo, and in vivo studies

2. Original language title.

For reviews in languages other than English, this field should be used to enter the title in the language of the review. This will be displayed together with the English language title.

3. * Anticipated or actual start date.

Give the date when the systematic review commenced, or is expected to commence.

09/02/2020

4. * Anticipated completion date.

Give the date by which the review is expected to be completed.

30/06/2020

5. * Stage of review at time of this submission.

Indicate the stage of progress of the review by ticking the relevant Started and Completed boxes. Additional information may be added in the free text box provided.

Please note: Reviews that have progressed beyond the point of completing data extraction at the time of initial registration are not eligible for inclusion in PROSPERO. Should evidence of incorrect status and/or completion date being supplied at the time of submission come to light, the content of the PROSPERO record will be removed leaving only the title and named contact details and a statement that inaccuracies in the stage of the review date had been identified.

This field should be updated when any amendments are made to a published record and on completion and publication of the review. If this field was pre-populated from the initial screening questions then you are not able to edit it until the record is published.

The review has not yet started: No

Review stage	Started	Completed
Preliminary searches	Yes	No
Piloting of the study selection process	Yes	No
Formal screening of search results against eligibility criteria	No	No
Data extraction	No	No
Risk of bias (quality) assessment	No	No
Data analysis	No	No

Provide any other relevant information about the stage of the review here (e.g. Funded proposal, protocol not yet finalised).

6. * Named contact.

The named contact acts as the guarantor for the accuracy of the information presented in the register record.

Eva Bongaerts

Email salutation (e.g. "Dr Smith" or "Joanne") for correspondence:
Eva

7. * Named contact email.

Give the electronic mail address of the named contact.

eva.bongaerts@uhasselt.be

8. Named contact address

PLEASE NOTE this information will be published in the PROSPERO record so please do not enter private information

Give the full postal address for the named contact.

Centre for Environmental Sciences, Hasselt University, Agoralaan Building D, 3590 Diepenbeek, Belgium

9. Named contact phone number.

Give the telephone number for the named contact, including international dialling code.

+3211268352

10. * Organisational affiliation of the review.

Full title of the organisational affiliations for this review and website address if available. This field may be completed as 'None' if the review is not affiliated to any organisation.

Hasselt University

Organisation web address:
https://www.uhasselt.be/en

11. * Review team members and their organisational affiliations.

Give the personal details and the organisational affiliations of each member of the review team. Affiliation refers to groups or organisations to which review team members belong. **NOTE: email and country are now mandatory fields for each person.**

Ms Eva Bongaerts. Hasselt University
Ms Thessa Van Pee. Hasselt University
Dr Hannelore Bové. Hasselt University
Professor Tim Nawrot. Hasselt University

12. * Funding sources/sponsors.

Give details of the individuals, organizations, groups or other legal entities who take responsibility for initiating, managing, sponsoring and/or financing the review. Include any unique identification numbers assigned to the review by the individuals or bodies listed.

Not applicable.

Grant number(s)

13. * Conflicts of interest.

List any conditions that could lead to actual or perceived undue influence on judgements concerning the main topic investigated in the review.

None

14. Collaborators.

Give the name and affiliation of any individuals or organisations who are working on the review but who are not listed as review team members. **NOTE: email and country are now mandatory fields for each person.**

15. * Review question.

State the question(s) to be addressed by the review, clearly and precisely. Review questions may be specific or broad. It may be appropriate to break very broad questions down into a series of related more specific questions. Questions may be framed or refined using PI(E)COS where relevant.

The goal of this systematic review is to combine all existing evidence on particle translocation during pregnancy and provide an answer to the question: “Can airborne (ultra)fine particles or engineered nanoparticles (NPs) transfer from mother to fetus during pregnancy?" In addition, we aim to discuss factors important for translocation and identify needs for further research.

16. * Searches.

State the sources that will be searched. Give the search dates, and any restrictions (e.g. language or publication period). Do NOT enter the full search strategy (it may be provided as a link or attachment.)

Different combinations of search terms related to “placenta”, “translocation” and “particles” will be used to construct Boolean operators. A literature search will be conducted by two independent reviewers, blinded to each other. To ensure literature saturation, we will screen the references in key review papers to find additional eligible publications that were not retrieved by our initial database search. In case of disagreement between the independent reviewers during the search process, a third reviewer will mediate. If multiple studies with overlapping study populations are identified, either the most recent or the most complete publication will be included. The searches will be re-run just before the final analyses to retrieve the most recent studies eligible for inclusion. No limit will be set on publication date; however, only articles reported in English will be included to avoid result misinterpretation due to incorrect translation. Only primary research will be included in this review.

17. URL to search strategy.

Give a link to a published pdf/word document detailing either the search strategy or an example of a search strategy for a specific database if available (including the keywords that will be used in the search strategies), or upload your search strategy.

Do NOT provide links to your search results.

https://www.crd.york.ac.uk/PROSPEROFILES/167478_STRATEGY_20200514.pdf

Do not make this file publicly available until the review is complete

18. * Condition or domain being studied.

Give a short description of the disease, condition or healthcare domain being studied. This could include health and wellbeing outcomes.

We want to gain insight into maternal-fetal particle translocation originating from environmental and occupational exposure.

19. * Participants/population.

Give summary criteria for the participants or populations being studied by the review. The preferred format includes details of both inclusion and exclusion criteria.

Human studies and animal studies relevant to human health are included here. In this systematic review, we focus on the translocation of particulate air pollutants and engineered nanoparticles (NPs) across the placental barrier in an *in vitro*

(human cell lines, *e.g.,* BeWo b30), *ex vivo* (*e.g.,* human placental perfusion model), and *in vivo* (animal models and human studies) context.

20. * Intervention(s), exposure(s).

Give full and clear descriptions or definitions of the nature of the interventions or the exposures to be reviewed.

Emphasis is placed on ambient air pollutants and engineered nanomaterials in particulate form, including, among others, particulate matter (PM) with particles ≤ 10 μm (PM10), particles ≤ 2.5 μm (PM2.5), ultrafine particles (UFP), and black carbon (BC). To maintain the focus on ambient air pollutants and engineered nanomaterials in particulate form, we exclude articles examining exposure to tobacco smoke, secondhand smoke, wildfire smoke, household or indoor sources of air pollution, PM chemical constituents or other volatile substances (*e.g.,* CO, O3, NO2, SO2), and nanomaterials characterized by a high aspect ratio (*e.g.,* nanotubes, nanosheets, or nanowires). In addition, therapeutic NPs were excluded since we want to maintain the focus on unintentional/environmental exposures.

21. * Comparator(s)/control.

Where relevant, give details of the alternatives against which the main subject/topic of the review will be compared (e.g. another intervention or a non-exposed control group). The preferred format includes details of both inclusion and exclusion criteria.

In this review, studies will be included in which comparisons are made between groups exposed to either a higher or a lower concentration of particles, as well as studies with a continuous exposure scale.

22. * Types of study to be included.

Give details of the types of study (study designs) eligible for inclusion in the review. If there are no restrictions on the types of study design eligible for inclusion, or certain study types are excluded, this should be stated. The preferred format includes details of both inclusion and exclusion criteria.

Eligible studies include human studies and animal studies relevant to human health
- *in vitro studies* (human cell lines, e.g. BeWo b30)
- *ex vivo* studies (*e.g.* human placental perfusion model)
- *in vivo* studies (animal or human studies)

that meet predefined inclusion criteria.

23. Context.

Give summary details of the setting and other relevant characteristics which help define the inclusion or exclusion criteria.

The placenta is a complex and transient organ that presents a natural barrier between mother and fetus while playing a crucial role in the growth, development and survival of the fetus. For a long time, it was believed that the placenta was an impenetrable barrier; however, it has been shown that multiple xenobiotics can bypass it. In recent years, several studies were conducted to investigate particle transfer across the placental barrier. These investigations are scarce and primarily consider in vitro cell cultures, ex vivo models and animal studies. Hence particulate translocation across the placental barrier following inhalation is insufficiently studied in a human context while being essential in understanding the effects on fetal health.

In this systematic review, emphasis will be placed on particulate air pollution, including PM10, PM2.5, UFP and BC particles, since prenatal air pollution exposure is associated with multiple adverse birth outcomes such as lower birth weight and premature birth. In addition, engineered nanomaterials are considered as well since exposure is increasing due to the rapidly emerging field of nanotechnology.

24. * Main outcome(s).

Give the pre-specified main (most important) outcomes of the review, including details of how the outcome is defined and measured and when these measurement are made, if these are part of the review inclusion criteria.

The primary outcome of this systematic review is the synthesis of all evidence regarding the maternal-fetal translocation of air pollution-related and engineered NPs.

* Measures of effect

Not applicable.

25. * Additional outcome(s).

List the pre-specified additional outcomes of the review, with a similar level of detail to that required for main outcomes. Where there are no additional outcomes please state 'None' or 'Not applicable' as appropriate to the review

Information about the number of particles detected per sample unit, translocation pathways, influencing factors and analytical methods used to quantify and characterize particle translocation will be provided along. Additionally, we aim to identify existing research gaps.

* Measures of effect

Not applicable.

26. * Data extraction (selection and coding).

Describe how studies will be selected for inclusion. State what data will be extracted or obtained. State how this will be done and recorded.

Two investigators will be appointed to conduct the literature search independently. They will screen all resulting titles and abstracts, and review full texts of articles that meet our predetermined inclusion criteria. Only articles considering exposure to one or more of the commonly measured air pollution particles or engineered NPs (PM2.5 (including BC), PM10, UFP, titanium dioxide NPs, silver NPs, etc.) and addressing their translocation across the placental barrier will be included. Besides human studies, the following studies relevant to human health will also be included; (i) *in vitro* studies across placental cellular barriers (*e.g.,* BeWo b30 cell line), (ii) *ex vivo* placental perfusion studies, and (iii) *in vivo* studies (*e.g.,* animal models). To maintain the focus on ambient air pollutants and engineered nanomaterials in particulate form, we exclude articles examining exposure to tobacco smoke, secondhand smoke, wildfire smoke, household or indoor sources of air pollution, PM chemical constituents or other volatile substances (*e.g.,* CO, O3, NO2, SO2), and nanomaterials characterized by a high aspect ratio (*e.g.,* nanotubes, nanosheets, or nanowires). No publication date restrictions will be adopted. We will exclusively include articles reported in English to avoid misinterpretation of results due to incorrect translation. Only original articles are considered as well. A study will be included when both reviewers independently define the full text as meeting the inclusion criteria. When there is a disagreement, a third reviewer will mediate.

Extracted data for each article will include; authors, model characteristics, experimental information (*i.e.,* nature of particle exposure, particle size, exposure route, exposure period, fetal unit under study, ...) and main findings (*i.e.,* degree of translocation, number of detected particles per sample unit, analytical methods to quantify and characterize particle translocation, influencing factors, …). If multiple studies with overlapping study populations are identified, either the most recent or the most complete publication will be included.

27. * Risk of bias (quality) assessment.

Describe the method of assessing risk of bias or quality assessment. State which characteristics of the studies will be assessed and any formal risk of bias tools that will be used.

The diversity in, among others, model, exposure route and particle dose, analytical detection method, etc. did not allow to carry out a risk of bias assessment.

28. * Strategy for data synthesis.

Provide details of the planned synthesis including a rationale for the methods selected. This **must not be generic text** but should be **specific to your review** and describe how the proposed analysis will be applied to your data.

Narrative systematic review without meta-analysis

29. * Analysis of subgroups or subsets.

State any planned investigation of 'subgroups'. Be clear and specific about which type of study or participant will be included in each group or covariate investigated. State the planned analytic approach.

Exposures will be grouped according to population (human or animal, further specified as *in vitro*, *in vivo,* or *ex vivo*). In addition, publications can be grouped into 'engineered nanoparticles' and 'particulate air pollution' referring to occupational and environmental exposure, respectively.

30. * Type and method of review.

Select the type of review and the review method from the lists below. Select the health area(s) of interest for your review.

Type of review

Cost effectiveness	No
Diagnostic	No
Epidemiologic	Yes
Individual patient data (IPD) meta-analysis	No
Intervention	No
Meta-analysis	No
Methodology	No
Narrative synthesis	Yes
Network meta-analysis	No
Pre-clinical	No
Prevention	No
Prognostic	No
Prospective meta-analysis (PMA)	No
Review of reviews	No
Service delivery	No
Synthesis of qualitative studies	No
Systematic review	Yes
Other	No

Health area of the review

Alcohol/substance misuse/abuse	No
Blood and immune system	No
Cancer	No
Cardiovascular	No
Care of the elderly	No
Child health	No
Complementary therapies	No
COVID-19	No
Crime and justice	No

Dental	No
Digestive system	No
Ear, nose and throat	No
Education	No
Endocrine and metabolic disorders	No
Eye disorders	No
General interest	No
Genetics	No
Health inequalities/health equity	No
Infections and infestations	No
International development	No
Mental health and behavioural conditions	No
Musculoskeletal	No
Neurological	No
Nursing	No
Obstetrics and gynaecology	No
Oral health	No
Palliative care	No
Perioperative care	No
Physiotherapy	No
Pregnancy and childbirth	Yes
Public health (including social determinants of health)	Yes
Rehabilitation	No
Respiratory disorders	No
Service delivery	No
Skin disorders	No
Social care	No
Surgery	No
Tropical Medicine	No

Urological	No
Wounds, injuries and accidents	No
Violence and abuse	No

31. Language.

Select each language individually to add it to the list below, use the bin icon to remove any added in error.

English

There is an English language summary.

32. * Country.

Select the country in which the review is being carried out from the drop down list. For multi-national collaborations select all the countries involved.

Belgium

33. Other registration details.

Give the name of any organisation where the systematic review title or protocol is registered (such as with The Campbell Collaboration, or The Joanna Briggs Institute) together with any unique identification number assigned. (N.B. Registration details for Cochrane protocols will be automatically entered). If extracted data will be stored and made available through a repository such as the Systematic Review Data Repository (SRDR), details and a link should be included here. If none, leave blank.

34. Reference and/or URL for published protocol.

Give the citation and link for the published protocol, if there is one

No I do not make this file publicly available until the review is complete

35. Dissemination plans.

Give brief details of plans for communicating essential messages from the review to the appropriate audiences.

Do you intend to publish the review on completion?

Yes

36. Keywords.

Give words or phrases that best describe the review. Separate keywords with a semicolon or new line. Keywords will help users find the review in the Register (the words do not appear in the public record but are included in searches). Be as specific and precise as possible. Avoid acronyms and abbreviations unless these are in wide use.

engineered; airborne; (ultra)fine particles; nanoparticles; pregnancy; placental barrier; maternal-fetal transfer.

37. Details of any existing review of the same topic by the same authors.

Give details of earlier versions of the systematic review if an update of an existing review is being registered, including full bibliographic reference if possible.

38. * Current review status.

Review status should be updated when the review is completed and when it is published. For newregistrations the review must be Ongoing.

Review_Completed_not_published

39. Any additional information.

Provide any other information the review team feel is relevant to the registration of the review.

40. Details of final report/publication(s).

This field should be left empty until details of the completed review are available.
