# Supplementary material for: Translocation of (ultra)fine particles and nanoparticles across the placenta; a systematic review on the evidence of in vitro, ex vivo, and in vivo studies
Source: Part Fibre Toxicol. 2020 Nov 2;17:56. doi: 10.1186/s12989-020-00386-8 (PMC7607677; doi:10.1186/s12989-020-00386-8)
Supplement: Supplementary file 2 — Additional file 2. Review methods description. [file 12989_2020_386_MOESM2_ESM.docx]

**Supplementary Material**

**Translocation of (ultra)fine particles and nanoparticles across the placenta; a systematic review on the evidence of *in vitro*, *ex vivo,* and *in vivo* studies**

**Eva Bongaerts^1^, Tim S. Nawrot^1,2^, Thessa Van Pee^1^, Marcel Ameloot^3^, Hannelore Bové^1,3^**^†^

1. Centre for Environmental Sciences, Hasselt University, Agoralaan Building D, 3590 Diepenbeek, Belgium.
2. Department of Public Health and Primary Care, KU Leuven, Herestraat 49 - box 703, 3000 Leuven, Belgium.
3. Biomedical Research Institute, Hasselt University, Agoralaan Building C, 3590 Diepenbeek, Belgium

^†^Address correspondence: Hannelore Bové, PhD, Centre for Environmental Sciences, Hasselt University, Agoralaan Building D, 3590 Diepenbeek, Belgium. Phone: +32 11 268381. Email: hannelore.bove@uhasselt.be

**Additional file 2: Review methods description**

**First stage: literature search**

A literature search was performed through two search engines (PubMed and Web of Science) using the combinations of terms described below.

- For the PubMed search**:**

("placenta"[MeSH Terms] OR "placent*"[Title/Abstract] OR "fetus"[Title/Abstract] OR "fetus"[MeSH Terms] OR "foetal"[Title/Abstract] OR ("umbilical"[Title/Abstract] AND "cord"[Title/Abstract])) AND ("transloc*"[Title/Abstract] OR "transfer"[Title/Abstract] OR "passage"[Title/Abstract] OR "penetrate"[Title/Abstract] OR "biodistribution"[Title/Abstract]) AND ("particl*"[Title/Abstract] OR "particulate*"[Title/Abstract] OR "nanoparticles"[Title/Abstract] OR "air pollution"[MeSH Terms] OR "air pollut*"[Title/Abstract] OR "particulate matter"[MeSH Terms] OR ("particul*"[Title/Abstract] AND "matter"[Title/Abstract]) OR "black carbon"[Title/Abstract] OR ("diesel"[Title/Abstract] AND "exhaust"[Title/Abstract]))

- For the Web of Science search:

(TS = ("placent*") OR TS = ("fetus") OR TS = ("foetal") OR TS = ("umbilical AND cord")) AND (TS = ("transloc*") OR TS = ("transfer") OR TS = ("passage") OR TS = ("transport") OR TS = ("penetrate") OR TS = ("biodistribution")) AND (TS = ("particl*") OR TS = ("particulate*") OR TS = ("nanoparticl*") OR TS = ("air pollution") OR TS = ("air pollut*") OR TS = ("particulate matter") OR TS = ("particul* AND matter") OR TS = ("black carbon") OR TS = ("diesel AND exhaust"))

Respectively, 296 and 351 references were retrieved in the PubMed and Web of Science searches.

All the references were exported to the citation manager Endnote. From a total of 647 references, removal of 105 duplicates left 542 unique citations. The reference lists of key review papers were manually screened to find additional eligible publications. In total, 29 (18 from reviews and 11 from other studies) additional articles were selected for full-text examination of eligibility.

**Second stage: screening of titles and abstracts**

For the 571 references, a table including authors, publication year, title, abstract, language, and kind of publication was built for screening.

The “Participants”, “Exposure,” “Comparator”, and “Outcomes” (PECO) statement reported below was developed to identify inclusion criteria for selecting studies relevant to answering our research question.

- *P*articipants: Human studies and animal studies relevant to human health. We focus on the translocation of particles across the placental barrier in an *in vitro* (human cell lines, *e.g.,* BeWo b30), *in vivo* (*e.g.,* mouse models, human studies), and *ex vivo* (*e.g.,* human placental perfusion model) context.
- *E*xposure: Exposure to outdoor air pollution particles and engineered nanomaterials
- *C*omparator: groups exposed to either a higher or a lower concentration of particles, as well as studies with a continuous exposure scale.
- *O*utcome: Particle translocation from mother to fetus of environmental particles

Eligibility was assessed using the following flow chart (Additional figure 1).

**Additional Figure 1** – Eligibility flowchart.
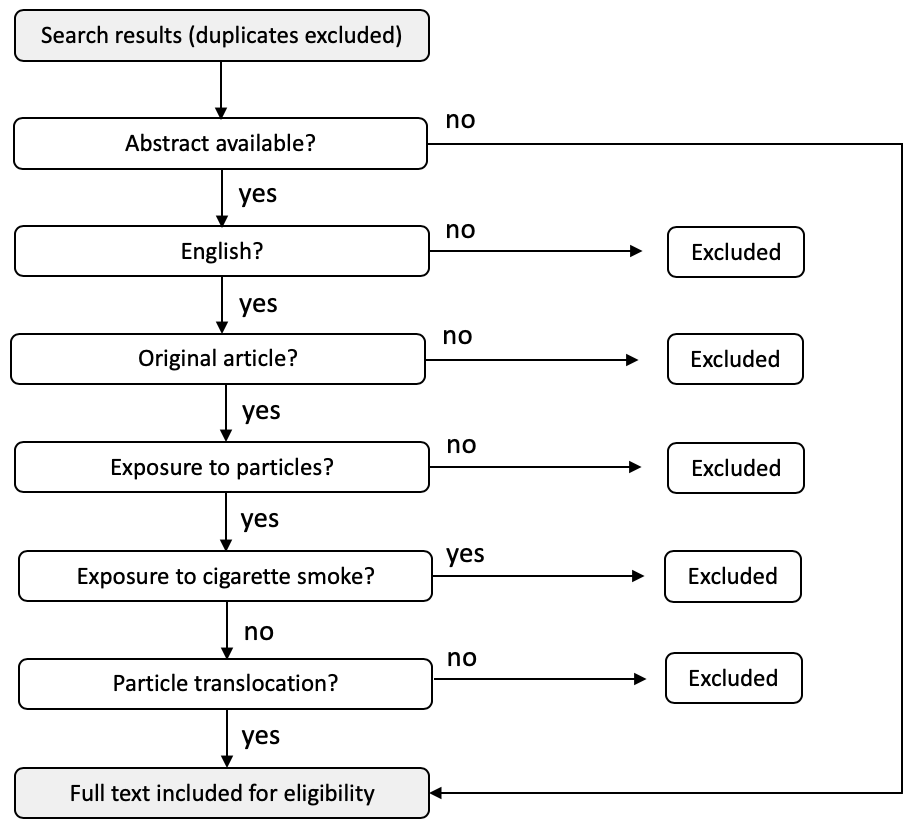


Among the articles screened, 463 were excluded and, in particular:

- 17 were not written in English,
- 72 were not original studies (51 reviews),
- 364 did not analyze particle exposure/translocation,
- 10 had smoking as the main exposure under study.

**Third stage: full-text examination of eligibility**

For the 108 references, full texts were searched to assess eligibility.

From the total of 108 articles, 35 were excluded from the next stage because:

- 2 had no full text available (even after contacting the authors),
- 9 did not measure translocation of particles across the placental barrier,
- 22 focused on therapeutic/diagnostic nanoparticles,
- 2 showed data already published in another study included in this review.

**Fourth stage: data extraction**

The final selection of 73 articles included:

- 21 studies on *in vitro* and/or *ex vivo* translocation in cell lines and placental perfusion models, respectively,
- 50 studies on particle translocation in animal models,
- 2 studies on particle translocation in humans.

For these final selected studies, the following information was extracted and summarized in tables: authors, population characteristics, experimental information (nature of particle exposure, particle size, exposure route, and study, the fetoplacental unit under study, etc.), the detection method of translocated particles, and main findings (degree of translocation, number of detected particles per sample unit, influencing factors, etc.).
